# Exploring the effects of palm kernel meal feeding on the meat quality and rumen microorganisms of Qinghai Tibetan sheep

**DOI:** 10.1002/fsn3.3340

**Published:** 2023-04-05

**Authors:** Ying Ma, Lijuan Han, Sayed Haidar Abbas Raza, Linsheng Gui, Xue Zhang, Shengzhen Hou, Shengnan Sun, Zhenzhen Yuan, Zhiyou Wang, Baochun Yang, Mohamed M. Hassan, Ruqaih S. Alghsham, Waleed Al Abdulmonem, Samia S. Alkhalil

**Affiliations:** ^1^ College of Agriculture and Animal Husbandry, Qinghai University Xining Xining 810016 People's Republic of China; ^2^ Guangdong Provincial Key Laboratory of Food Quality and Safety/Nation‐Local Joint Engineering Research Center for Machining and Safety of Livestock and Poultry Products South China Agricultural University Guangzhou 510642 China; ^3^ College of Animal Science and Technology, Northwest A&F University Yangling 712100 Shaanxi People's Republic of China; ^4^ Department of Biology College of Science, Taif University P.O. Box 11099 Taif 21944 Saudi Arabia; ^5^ Department of Pathology College of Medicine, Qassim University Qassim Saudi Arabia; ^6^ Department of Pathology College of Medicine, Qassim University P.O. Box 6655 Buraidah 51452 Kingdom of Saudi Arabia; ^7^ Department of Clinical Laboratory Sciences College of Applied Medical Sciences, Shaqra University Alquwayiyah Riyadh Saudi Arabia

**Keywords:** meat quality, palm kernel meal, rumen microorganisms, Tibetan sheep

## Abstract

Palm kernel meal (PKM) has been shown to be a high‐quality protein source in ruminant feeds. This study focused on the effects of feed, supplemented with different amounts of PKM (ZL‐0 as blank group, and ZL‐15, ZL‐18, and ZL‐21 as treatment group), on the quality and flavor profile of Tibetan sheep meat. Furthermore, the deposition of beneficial metabolites in Tibetan sheep and the composition of rumen microorganisms on underlying regulatory mechanisms of meat quality were studied based on ultra‐performance liquid chromatography coupled with quadrupole time‐of‐flight mass spectrometry as well as 16S rDNA sequencing. The results of the study showed that Tibetan sheep in the ZL‐18 group exhibited superior eating quality and flavor profile while depositing more protein and fat relative to the other groups. The ZL‐18 group also changed significantly in terms of the concentration and metabolic pathways of meat metabolites, as revealed by metabolomics. Metabolomics and correlation analyses finally showed that PKM feed mainly affected carbohydrate metabolism in muscle, which in turn affects meat pH, tenderness, and flavor. In addition, 18% of PKM increased the abundance of *Christensenellaceae R‐7 group, Ruminococcaceae UCG‐013, Lachnospiraceae UCG‐002*, and *Family XIII AD3011 group* in the rumen but decreased the abundance of *Prevotella 1*; the above bacteria groups regulate meat quality by regulating rumen metabolites (succinic acid, DL‐glutamic acid, etc.). Overall, the addition of PKM may improve the quality and flavor of the meat by affecting muscle metabolism and microorganisms in the rumen.

## Graphical abstract

18% of PKM group exhibited superior eating quality and flavor profile while depositing more protein and fat relative to the other group. A total of 23 differential metabolites were identified in PKM‐treated Tibetan sheep meat. Carbohydrate metabolism was typical metabolic pathway altered by PKM feeding. PKM feeding affected key metabolites by altering rumen microorganisms to regulate the relevant metabolic pathways

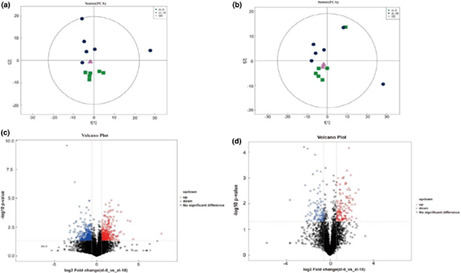

## 1 INTRODUCTION

In addition to its unique resources, the Qinghai‐Tibetan Plateau also represents one of the three major areas of livestock production in China (Li et al., 2021; Wen et al., 2014). Tibetan sheep, a characteristic livestock species of this plateau, are important for the region's ecology as well as the livelihood of Tibetan herders (Ma et al., 2022). Traditionally, the feeding mode of Tibetan sheep has been largely based on grazing, but with the increasing demand for animal products and the need to develop an ecological system of animal husbandry, the breeding mode has gradually changed toward semi‐herding and housing (Ma et al., 2022; Zhang, Han, Hou, Raza, Wang, et al., 2022). This gradual transition to indoor feeding systems has subsequently increased the need for vegetable protein feeds for livestock. In this context, soybean meal (SBM) represents a commonly used protein supplement in commercially available feeds, but a continuous rise in the price of SBM has been increasing feed costs significantly (Phulia et al., 2017). Given that alternative sources of cheaper protein will likely reduce the cost of livestock (Khan et al., 2016), animal nutritionists have been exploring the potential of using natural substances as a substitute for protein sources.

PKM, a by‐product of palm kernel oil extraction, is produced in large amounts during palm oil production (Corley, 2009). Its proteins, for which the content ranges from 12% to 21%, are not only of ideal quality but are also at an appropriate level for infants, as reported by WHO (Faridah et al., 2020; Sundu et al., 2006). In addition, the crude fiber (CF) content of PKM ranges from 16% to 18%, which is acceptable for most ruminants (Alshelmani et al., 2016). PKM can also be used as a source of calcium, manganese, zinc, and sodium in ruminant feeds (Akinyeye, 2011), with some studies even reporting that it contains large amounts of non‐starch polysaccharides (NSP), including 78% of linear mannans, 12% of cellulose, 3% of glucuronide xylan, and 3% of arabinoxylan (Sundu et al., 2006). Mannan is further divided into glucomannan, galactomannan, and galactoglucomannan according to the type of sugar units in its sugar chain (Moreira, 2008), while galactomannan, as a soluble NSP, can delay the passage of surimi in the animal intestine to ensure optimal absorption of nutrients and safeguard the intestinal health and physical development of animals (Mateos et al., 2012). So far, many scholars have added PKM to different feeds such as those of pigs, goats, and broilers. Jaworski et al. (2014) found that the addition of 15% PKM to the diet of weaned piglets did not affect their overall growth performance. Silva et al. (2020) found that adding 115 g kg of PKM to a high‐concentration diet raised in goats increased carcass weight and increased consumer acceptance of goat meat. Alshelmani et al. (2016) found that PKM fermented by *Bacillus polymyxa* ATCC 842 at 15% addition improved the intestinal flora of broilers and had no effect on nutrient digestibility. These progress has proven the feasibility of using PKM as feed, including for ruminants (Azizi et al., 2021).

Therefore, feeds that use protein components other than soybean meal are currently being investigated. For example, cottonseed meal can be an important substitute for soybean meal, especially since it has no negative effects on the performance of lambs (Silva et al., 2016). Similarly, using peanut meal as a substitute in feeds can improve the fatty acid profile of sheep meat (Lima Valença et al., 2020), while flaxseed meal can effectively improve sheep performance as well as the efficiency of feed utilization (Hao et al., 2020). PKM can also be used instead of soybean meal due to its secondary effects on the growth performance of goats (Silva et al., 2020). These examples clearly illustrate the effects of protein feed on sheep meat. This is because in animals, different feeds affect the deposition of muscle metabolites (Liu et al., 2022; Su et al., 2022; Wang, Zhao, et al., 2022), with the latter not only reflecting the animals' physiological conditions but also influencing the entire spectrum of meat‐related compounds. In addition, feed, a key factor that affects the composition and functions of rumen microbes in ruminants, alters the rumen's environment and metabolism (Olijhoek et al., 2022; Zhao et al., 2022). This process subsequently alters the deposition of metabolites in muscles. For example, a Dioscorea opposite waste diet in sheep increased the relative abundance of *Succiniclasticum* and *Ruminococcus_1* which enhanced rumen fermentation and average daily body weight (Guo et al., 2022). At the same time, *Succiniclasticum* is largely involved in the fermentation of succinic acid to propionic acid, with the latter being the most important precursor of glucose in ruminants. Consequently, in this study, it was hypothesized that PKM could also alter the rumen microorganism composition of Tibetan sheep to affect muscle metabolism and meat quality.

There is a lack of knowledge regarding the mechanisms through which PKM can regulate meat quality in Tibetan sheep, despite the fact that protein feeding is a key trait that determines quality. Therefore, to investigate how the addition of PKM to feed influences the meat quality and flavor of Tibetan sheep, the significant effects of PKM on the composition of rumen microbiota as well as the latter's relationship to muscle metabolism were studied using 16S rDNA sequencing and metabolomics. It is expected that the results of this study will help to identify the optimum level of palm meal that can be added to Tibetan sheep feed to save feed costs while promoting the development of this livestock industry in Qinghai.

## 2 MATERIALS AND METHODS

### 2.1 Ethics statement

All animal procedures for experiments were approved by the Committee of Experimental Animal Care, and handling techniques were approved (QUA‐2020‐0709) by the Qinghai University of Animal Care Committee.

### 2.2 Tibetan sheep and meat samples

In this study, one hundred and twenty 2‐ to 3‐month‐old healthy Qinghai Tibetan sheep, with similar body conditions (18.00 ± 3.00 kg), were selected. During the initial acclimatization phase, all animals were fed native pasture grass and provided with access to water on a daily basis. After the 14‐day adaptation period, the 120 animals were randomly divided into three treatment groups (ZL‐15, ZL‐18, and ZL‐21) and one control group (ZL‐0), as shown in Table 1. Each group contained 30 sheep and were placed in separate sheepfolds. In addition, the nutritional ingredient of PKM was determined, including 7.90% moisture, 4.60% crude ash, 16.50% crude protein, 31.10% crude fiber, 6.00% crude fat, 33.20% acid detergent fiber, and 72.20% neutral detergent fiber.

**TABLE 1:** Composition and nutritional level of concentrate supplements in diets of sheep.

	ZL‐0	ZL‐15	ZL‐18	ZL‐21
Raw material composition/%				
Maize	65.5	54.20	51.80	49.50
Soybean meal	8.00	5.00	5.00	4.00
Rapeseed meal	16.00	15.00	15.00	15.00
Cottonseed meal	4.00	4.30	3.70	4.00
PKM	0.00	15.00	18.00	21.00
Common salt	0.50	0.50	0.50	0.50
Mountain flour	1.00	1.00	1.00	1.00
Premix	5.00	5.00	5.00	5.00
Nutritional ingredient/%				
Crude protein	15.15	15.12	15.13	15.13
Crude fat	2.70	3.42	3.58	3.72
Crude fiber	3.83	5.78	6.16	6.58
Lysine	0.66	0.63	0.63	0.63
Methionine	0.26	0.28	0.28	0.28
Calcium	0.54	0.52	0.51	0.51
Phosphorus	0.43	0.38	0.36	0.35

The fattening period of lambs was 90 days, with their diet composition at different experimental stages (calculated by dry matter) shown in Table 2. The four groups of sheep were all housed and fed separately. In this case, each sheepfold was equipped with a sports field, and the enclosure was sunny, dry, and well ventilated, while being sheltered from wind. The lambs were fed twice daily at 8:30 a.m. and 4:30 p.m. with free access to food and water. After the feeding period, five Tibetan sheep were randomly selected from each group and transported to a commercial slaughterhouse where their carotid artery and jugular vein were cut off after mechanical electric shock (fasting and water fasting for 12 h before slaughter). The *longissimus lumborum* (LL) was then collected from the 9th to 11th ribs on the back of the carcass. Meanwhile, the rumen contents were collected and filtered to obtain the filtrate. All slaughter and sampling were professionally performed according to uniform standards, with the collected samples subsequently stored at −80°C until required for analyses.

**TABLE 2:** Diet composition of lambs at different experimental stages (calculated by dry matter).

Driving cycle	Supplementary concentrate (%)	Silage oat grass (%)	Dry oat grass (%)
Phase I (first 30 days)	60	20	20
Phase 2 (60 days later)	70	15	15

### 2.3 Analysis of the carcass traits of meat

Live weight was measured and recorded 12 h before slaughter. After slaughter, the skin, head, hoof, tail, and viscera were removed, and the body weight of the plate oil and kidney was retained as the carcass weight. After 24 h of slaughter, the cross‐sectional area of the carcass between the 12th and 13th calcaneus ribs was drawn on the hydrochloric acid paper, and the surface area was calculated by AutoCAD. The result was the eye muscle area. At the same time, the thickness of rib meat between 12th and 13th heel ribs was measured and recorded as rib meat thickness. Backfat thickness was measured by Vernier caliper between the 3rd and 4th ribs at 6–8 cm from the midline (Chen et al., 2022; Devapriya et al., 2021; Güngör et al., 2022).

### 2.4 Analysis of the edible quality and nutritional component of meat

The pH_45 min_ and pH_24 h_ were determined as described by Zhang, Guo, Su, Corazzin, Hou, et al. (2022), after the Tibetan sheep had been left for 45 min and 24 h after slaughter, respectively. The color parameters, namely the brightness (L*), redness (a*), and yellowness (b*), were also evaluated according to the method of Zhang, Han, Hou, Raza, Wang, et al. (2022), using a calibrated automated colorimeter (TCP_2_‐AE/BE/CE, Kett Japan Kite).

Before determining the edible and nutritional quality of LL muscles, the samples were removed from the −80°C refrigerator, placed in sealed bags, and slowly thawed at 4°C. After complete thawing, the samples were split for subsequent testing. The steam loss rate and cooked meat rate were determined using LL pieces of 5 cm × 1 cm × 1 cm (length, width, and height). To measure the shear force, the samples were first cut into 3 cm × 1 cm × 1 cm meat columns in the direction of the muscle fibers, using an iron ruler and a scalpel. A muscle tenderness meter was then used in the direction of the vertical muscle fiber. Finally, the physical properties (hardness, elasticity, and chewiness) were determined using the TPA mode (CT3 10 K, United States) after cutting the samples into pieces of 1 cm × 1 cm × 1 cm.

To assess the nutritional composition of LL muscles, the thawed meats were churned in a meat grinder before determining their moisture, protein, and fat content using the Analytical Chemists Association's (AOAC, 2000) Standards.

### 2.5 Analysis of amino acid and fatty acid composition of meat

For amino acid (AA) analysis, an Agilent 1290 Infinity LC ultra‐high‐performance liquid chromatography system (Agilent, Waldbronn, Germany) was used along with a 5500 QTRAP mass spectrometer (AB SCIEX). The conditions used for the high‐performance liquid chromatography and mass spectrometry were as described by Zhang, Han, Hou, Raza, Gui, et al. (2022). The HPLC conditions were as follows: the mobile phase: 25 mM ammonium format +0.08% FA aqueous solution for liquid A and 0.1% FA acetonitrile for liquid B. The sample was injected into an autosampler at 4°C with a flow rate of 250 μL/min and an injection volume of 1 μL. The sample was injected into the autosampler at 4°C with a column temperature of 40°C, a flow rate of 250 μL/min, and an injection volume of 1 μL. The relevant liquid phase gradients were as follows: 0–12 min, liquid B changed linearly from 90% to 70%; 12–18 min, liquid B changed linearly from 70% to 50%; 18–25 min, liquid B changed linearly from 50% to 40%; 30–30.1 min, B‐fluid changed linearly from 40% to 90%; and 30.1–37 min, B‐fluid was maintained at 90%. QC sample was set up for every certain number of experimental samples in the sample cohort to test and evaluate the stability and reproducibility of the system. The 5500 QTRAP ESI source conditions are as follows: source temperature 500°C, ion Source Gas1 (Gas1): 40, Ion Source Gas2 (Gas2): 40, Curtain gas (CUR): 30, ionSapary Voltage Floating (ISVF): 5500 V; and MRM mode was used to detect the ion pair to be measured pairs.

To determine the fatty acid (FA) composition, samples were first separated by gas chromatography performed on an Agilent DB‐WAX column (30 m × 0.25 mm ID × 0.25 mm). Mass spectrometry analysis was then carried out using an Agilent 7890/5975C gas‐mass spectrometer (Agilent, Waldbronn, Germany). For both steps, the methods of Zhang, Han, Hou, Raza, Gui, et al. (2022) and Santos et al. (2022) were followed. The specific gas chromatographic conditions were as follows: programmed ramp‐up: initial temperature 50°C; hold for 3 min; ramp‐up to 220°C at 10°C/min; and maintain for 5 min. The carrier gas was helium at a flow rate of 1.0 mL/min. QC sample was set up for each number of experimental samples in the sample cohort, which was used to test and evaluate the stability and performance of the system. The mass spectrometry conditions were as follows: inlet temperature 280°C; ion source temperature 230°C; transmission line temperature 250°C; and electron bombardment ionization (EI) source, SIM scan mode, and electron energy 70 eV.

### 2.6 Analysis of volatile flavor components (VOCs) of meat

Two grams of LL sample were transferred to a 20‐mL headspace vial for GC‐IMS (gas chromatography‐ion mobility spectrometry) analysis using the FlavourSpec® (GAS) flavor analyzer. Two‐gram sample was thawed in a refrigerator at 4°C for 12 h and loaded into a 20 mL headspace vial, which was incubated by preheating at 80°C for 15 min and subsequently pumped into the injector with a 500 μL headspace volume at 85°C. The temperature of the chromatographic column was fixed at 60°C, and the temperature of the drift tube was kept at 45°C. The flow rate of the carrier gas nitrogen (99.999% purity) was 150 mL/min (constant flow rate) and the flow rate of the GC column was 2 mL /min for 2 min. The flow rate was then increased to 20 mL/min for 8 min and further increased to 100 mL/min for 15 min before stopping. VOCs were determined by comparing retention indices (RI) and drift times (Dt) of standards from the NIST and IMS databases. The proportion of each component in the meat samples was estimated by normalized quantitative analysis of the peak volume of each component separately.

### 2.7 Analysis of non‐target metabolites of meat

After thawing LL muscle samples slowly at 4°C, 80 mg was homogenized and vortexed in 200 L of water for 60 s. Methanol acetonitrile solution (pre‐cooled; 800 μL) was then added in a 1:1 ratio prior to vortexing and sonication at low temperature for 30 min. The samples were then kept at −20°C for 1 h and centrifuged for 20 min at 4°C. The resulting supernatant was dried under vacuum and eventually used for ultra‐performance liquid chromatography and quadrupole time‐of‐flight mass spectrometry (UHPLC‐QTOF‐MS) analysis.

In order to separate the metabolites, an Agilent 1290 Infinity LC ultra‐high‐performance liquid chromatography system (UHPLC) was used. In this case, a column temperature of 25°C and a torch flow rate of 0.3 mL/min were applied. For mobile phase A, water and 25 mM of ammonium acetate were used, while mobile phase B consisted of acetonitrile. Samples were placed in a 4°C autosampler throughout the analysis and were also analyzed in a random order in order to avoid the effects of fluctuations in the instrument's detection signal. In addition, to monitor and evaluate the stability of the system as well as the reliability of the experimental data, QC samples were inserted into the sample queue. Primary and secondary spectra of the samples were acquired using the AB Triple TOF 6600 mass spectrometer, with the ESI conditions applied as described by Zhang, Han, Hou, Raza, Wang, et al. (2022).

The raw data were converted by ProteoWizard into an mzXML format (MetDDaA and LipDDA identified metabolite structures) and then aligned. They were also corrected for retention time before extracting the peak areas by XCMS. After being normalized to total peak intensity, the processed data were imported into SIMCA‐P (version 14.1, Umetrics) where differential metabolites were identified prior to KEGG pathway analysis (Kyoto Encyclopedia of Genes and Genomes, http://www.kegg.jp/).

### 2.8 Quantification of rumen microflora and analysis of bacterial diversity

Rumen content collected from Tibetan sheep was filtered through multiple layers of gauze to obtain filtrates which were then preserved in liquid nitrogen until required for subsequent analysis. Total genomic DNA was extracted from the samples using the CTAB/SDS method prior to PCR amplification of the V3–V4 regions of the 16S rDNA according to specific barcode primers. AxyPrepDNA Gel Extraction kit (AXYGEN) was then used to purify mixed PCR products, with required fragments subsequently quantified by electrophoresis using the QuantiFluor^TM^‐STBlue Fluorescence Quantification System. In this case, the mixing ratio was based on the amount of sequencing required for each sample. The NEB Next® Ultra^TM^ DNA Library Prep Kit was subsequently used for library preparation, and after passing quality control, the libraries were analyzed on an Agilent Bioanalyzer 2100 and Qubit. Finally, the library was sequenced on the Illumina Miseq platform to generate 250 bp paired‐end reads.

The raw data obtained by sequencing were cleaned by splicing and filtering the sequences before removing chimeras in order to enhance the accuracy and reliability of the reads. Sequences were then clustered into OTUs (operational taxonomic units) with 97% agreement before being classified based on valid data (Caporaso et al., 2011; Youssef et al., 2009). The results were then used to analyze various diversity indicators and detect sequencing depth. In addition, statistical analyses of community structures were performed at each taxonomic level (DeSantis Jr. et al., 2006; Hess et al., 2011; Lozupone et al., 2005).

### 2.9 Statistical analysis and network analysis

Data on muscle quality (eating quality, nutritional quality, AA, FA, and VOCs), muscle metabolism, and rumen microbiota composition of Tibetan sheep which were given different levels of PKM diets were compared using one‐way ANOVA on IBM SPSS 23 software. Results were then expressed as means and standard error of means (SEM), and considered to be statistically significant when *p*‐values were <.05. The relationship among meat quality, muscle metabolism, and rumen bacteria was also determined using Pearson's correlation coefficient. In this case, results for which *p*‐values were <.05 and |*r*| values were >.50 were considered to be statistically significant.

## 3 RESULTS

### 3.1 The effects of PKM on the carcass traits

Carcass traits are the most important indicators to measure the economic value of a meat sheep, and they are also the most important part of meat sheep performance measurement, including pre‐slaughter weight, carcass weight, slaughter rate, rib meat thickness, backfat thickness, and eye muscle area. As shown in Table 3, there was no significant difference in carcass traits of Tibetan sheep after adding PKM to the feed. However, the live weight of ZL‐21 was significantly lower than that of ZL‐0 (*p* < .05).

**TABLE 3:** Effects of different levels of palm meal added in feed on the carcass traits of Tibetan sheep meat.

Index	ZL‐0	Zl‐15	Zl‐18	Zl‐21
Live weight/kg	34.97 ± 2.55^a^	33.14 ± 3.16^ab^	32.92 ± 3.71^ab^	31.23 ± 3.21^b^
Carcass weight/kg	16.58 ± 0.97	17.30 ± 0.84	17.44 ± 1.52	16.52 ± 1.62
Dressing percentage/%	47.41 ± 2.77	52.20 ± 2.53	52.98 ± 4.63	52.90 ± 5.18
Loin eye area/cm^2^	18.17 ± 2.22	18.30 ± 4.16	21.66 ± 1.96	19.19 ± 3.24
Rib meat thickness/cm	5.57 ± 0.72	5.84 ± 1.15	7.39 ± 0.84	6.77 ± 0.16
Backfat thickness/cm	6.10 ± 0.73	5.20 ± 1.26	7.06 ± 0.64	7.59 ± 0.31

*Note*: The same lowercase letters or no letters indicate no significant difference (*p* > .05), and different lowercase letters indicate significant difference (*p* < .05).

### 3.2 The effects of PKM on the edible quality and nutritional component

The edible quality of meat mainly refers to its color, texture, tenderness, and water retention capacity. As shown in Table 4, the pH of meat at 45 min was significantly lower after adding 18% (ZL‐18) PKM to the feed (*p* < .05). However, compared with the other three groups, the pH was significantly higher after 24 h of acid discharge with ZL‐0 group (*p* < .05). In addition, for the a* values, those of ZL‐18 and ZL‐21 were significantly higher compared with ZL‐15 (*p* < .05). In terms of the water retention of meat, the cooking loss rate of ZL‐21 was significantly higher than that of the other three groups, while its cooked meat rate was significantly lower than for ZL‐15 (*p* < .05). Finally, Tibetan mutton displayed better meat tenderness after being fed with PKM diet, with the shear force and chewiness of ZL‐18 being the smallest (*p* < .05).

**TABLE 4:** Effects of different levels of palm meal added in feed on the edible quality and nutritional component of Tibetan sheep meat.

Index	ZL‐0	Zl‐15	Zl‐18	Zl‐21
pH_45 min_	5.93 ± 0.19^a^	6.01 ± 0.21^a^	5.67 ± 0.13^b^	5.95 ± 018^a^
pH_24 h_	5.63 ± 0.05^a^	5.56 ± 0.05^b^	5.56 ± 0.08^b^	5.55 ± 0.09^b^
L*	31.79 ± 1.41	31.33 ± 1.33	32.94 ± 1.74	32.32 ± 2.93
a*	11.05 ± 2.30^bc^	9.72 ± 2.71^c^	13.25 ± 3.25^ab^	15.55 ± 2.19^a^
b*	12.63 ± 2.16	11.48 ± 2.81	12.50 ± 2.46	13.57 ± 2.89
Cooking loss	28.95 ± 2.93^b^	29.32 ± 3.40^b^	29.88 ± 3.11^b^	34.39 ± 2.68^a^
Cooking percentage	66.97 ± 3.11^ab^	67.92 ± 3.45^a^	67.44 ± 3.05^ab^	64.62 ± 3.30^b^
Tenderness	30.58 ± 1.82^a^	27.47 ± 1.49^b^	26.41 ± 0.83^b^	32.80 ± 1.64^a^
Hardness	4716.22 ± 495.95	4664.52 ± 616.93	4734.26 ± 298.20	4950.54 ± 264.40
Elastic	0.26 ± 0.03	0.28 ± 0.02	0.28 ± 0.03	0.28 ± 0.02
Chewiness	37.56 ± 1.55^a^	35.89 ± 1.22^ab^	34.90 ± 1.38^b^	37.50 ± 1.28^a^
Water	74.70 ± 1.78	75.64 ± 1.33	74.83 ± 1.30	74.78 ± 1.46
Protein	21.13 ± 0.99c	21.85 ± 2.39bc	22.82 ± 1.89ab	23.47 ± 0.96a
Fat	3.82 ± 0.63b	4.01 ± 0.44ab	4.41 ± 0.71a	4.14 ± 0.56ab

*Note*: The same lowercase letters or no letters indicate no significant difference (*p* > .05), and different lowercase letters indicate significant difference (*p* < .05). ZL‐0: PKM was added to feed at 0%, ZL‐15: PKM was added to feed at 15%, ZL‐18: PKM was added to feed at 18%, and ZL‐21: PKM was added to feed at 21%.

Fat, protein, and water content are usually measured to determine the nutritional quality of meat. As shown in Table 4, compared with the blank group (ZL‐0), the amount of protein and fat in Tibetan mutton increased after adding PKM to the feed. More specifically, ZL‐18 and ZL‐21 had significantly higher protein and fat content compared with ZL‐0 (*p* < .05), hence indicating that the muscles of Tibetan sheep could effectively deposit protein and fat after being given protein feed.

### 3.3 The effects of PKM on the AA profiles and FA profiles

The effects of PKM feed on amino acid composition and content are shown in Table 5.

**TABLE 5:** Effects of different levels of palm meal added in feed on the AA profiles of Tibetan sheep meat (umol/g).

Index	ZL‐0	Zl‐15	Zl‐18	Zl‐21
TAA	37.91 ± 4.50	35.36 ± 0.73	36.30 ± 2.37	32.86 ± 2.04
EAA	3.36 ± 0.25	3.23 ± 0.14	3.28 ± 0.36	3.05 ± 0.09
NEAA	34.55 ± 4.28	32.13 ± 0.73	33.03 ± 2.24	29.80 ± 1.97

*Note*: ZL‐0: PKM was added to feed at 0%, ZL‐15: PKM was added to feed at 15%, ZL‐18: PKM was added to feed at 18%, and ZL‐21: PKM was added to feed at 21%.

Abbreviations: EAAs, essential amino acids; NEAAs: non‐essential amino acids; TAAs, total amino acids.

Table S1. There were no significant differences in total amino acids (TAAs), essential amino acids (EAAs), and non‐essential amino acids (NEAAs) among the four groups' LL.

In terms of fatty acids, saturated fatty acids and monounsaturated fatty acids contribute mainly to meat flavor, whereas polyunsaturated ones contribute to meat nutrition. There are different types of fatty acids in Tibetan mutton, and in this study, 35 (mainly oleic acid, stearic acid, and palmitic acid) were detected in the samples. As shown in Table 6, the amount of saturated fatty acid (SFA) in ZL‐18 was the highest (*p* < .05), while those of lauric acid, myristic acid, and palmitic acid were also significantly higher compared with the other three groups (*p* < .05). Lauric acid accounted for a large proportion of SFA in Tibetan mutton. However, the amount of neuronic acid also increased significantly after feeding PKM (*p* < .05).

**TABLE 6:** Effects of different levels of palm meal added in feed on the FA profiles of Tibetan sheep meat (ug/g).

Index	ZL‐0	Zl‐15	Zl‐18	Zl‐21
C11:0	0.06 ± 0.04^ab^	0.04 ± 0.04^b^	0.15 ± 0.05^a^	0.06 ± 0.02^ab^
C12:0	4.60 ± 2.76^b^	6.49 ± 2.43^ab^	15.23 ± 6.47^a^	6.66 ± 2.25^ab^
C14:0	166.80 ± 107.03^b^	194.84 ± 67.88^b^	449.18 ± 53.32^a^	195.48 ± 97.05^b^
C15:0	17.72 ± 9.83^ab^	15.01 ± 3.01^b^	31.63 ± 5.35^a^	15.06 ± 2.41^ab^
C16:0	2109.82 ± 814.99^b^	2238.02 ± 609.37^b^	3673.17 ± 260.54^a^	2192.47 ± 700.89^b^
C24:1 N9	0.84 ± 0.03^b^	1.13 ± 0.08^a^	1.16 ± 0.09^a^	1.27 ± 0.07^a^
SFA	3832.04 ± 1657.44^b^	4074.24 ± 1319.65^b^	6561.62 ± 757.20^a^	3800.64 ± 1108.73^b^
MUFA	3992.42 ± 1652.05	4235.45 ± 1360.88	5848.97 ± 515.75	4264.62 ± 1193.48
PUFA	853.03 ± 89.78	813.15 ± 75.49	905.64 ± 251.57	860.48 ± 37.46
N3	95.41 ± 7.38	103.12 ± 18.62	112.27 ± 28.60	106.12 ± 13.88
N6	751.62 ± 82.40	710.04 ± 57.04	793.37 ± 222.97	754.37 ± 34.09

*Note*: N3: sum of omega‐3 polyunsaturated fatty acids (18:3n‐3, 20:3n‐3, 20:5n‐3, 22:5n‐3, and 22:6n‐3); N6: sum of omega‐6 polyunsaturated fatty acids (18:2n‐6, 18:3n‐6, 20:3n‐6, 20:4n‐6, 22:2n‐6, 22:4n‐6, and 22:5n‐6). ZL‐0: PKM was added to feed at 0%, ZL‐15: PKM was added to feed at 15%, ZL‐18: PKM was added to feed at 18%, and ZL‐21: PKM was added to feed at 21%.

Abbreviations: MUFA, sum of monounsaturated fatty acids; PUFA, sum of polyunsaturated fatty acids; SFA, sum of saturated fatty acid.

### 3.4 The effects of PKM on the volatile flavor components

**FIGURE 1:**
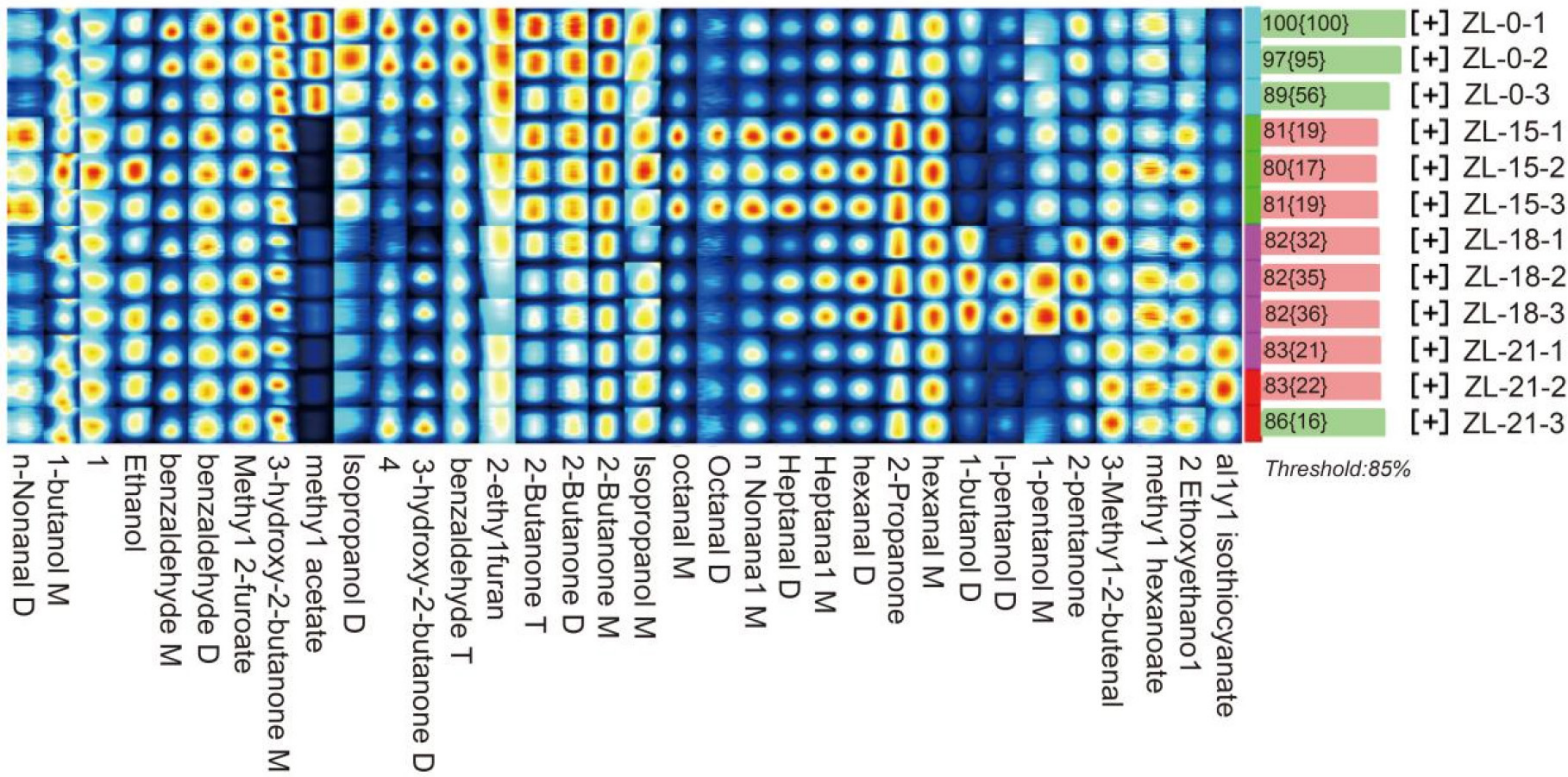
Fingerprinting of volatile flavor substances in four groups of Tibetan sheep samples, ZL‐0: PKM was added to feed at 0%, ZL‐15: PKM was added to feed at 15%, ZL‐18: PKM was added to feed at 18%, and ZL‐21: PKM was added to feed at 21%.

Flavor can determine the quality of sheep meat. To examine the effects of different levels of PKM on the production of VOCs in Tibetan sheep muscle, GC‐IMS was used. A total of 32 volatile substances, including 12 aldehydes, 8 alcohols, 7 ketones, 4 esters, and 2‐ethylfuran, were detected in the four groups of Tibetan lamb. Figure 1 shows that the fingerprints of the Tibetan sheep meat were formed from the peak signals. By comparing fingerprints between samples, volatile organic compounds could then be dynamically monitored over time. In this case, changes (color depth represents signal strength) in flavor between different groups were determined by comparing the intensity of distribution for the volatile compounds. Methyl acetate, isopropanol, 3‐hydroxy‐2‐butanone, benzaldehyde, 2‐ethylfuran, and 2‐butanone were detected at higher levels in the control group, although their levels decreased with the addition of PKM to the feed. In contrast, levels of 3‐methyl‐2‐butenal, methyl hexanoate, 2‐ethoxyethanol, and allyl isothiocyanate increased slightly with increasing PKM content in the feed. The relative proportions of VOCs in each group are also shown in Table 7. In this case, the addition of PKM to the feed significantly increased the proportions of aldehydes in the muscle, especially compared with ZL‐0, ZL‐15, and ZL‐21 for which significantly higher levels of non‐Nonanal D, M, and octanal M were obtained (*p* < .05). Furthermore, ZL‐15 and ZL‐18 also showed significantly higher levels of hepanal D, hepanal M, heptanal D, and heptanal M (*p* < .05). The addition of 18% PKM to the feed significantly reduced the proportion of isopropanol D and 3‐hydroxy‐2‐butanone M in the muscle, while significantly increasing the proportion of 1‐butanol D, 2‐ethoxyethanol, 2‐propanone, and 2‐pentanone (*p* < .05). The proportion of 3‐hydroxy‐2‐butanone D and methyl acetate decreased after the addition of 15% PKM to the feed (*p* < .05). It should further be noted that there was an overall decrease in the proportion of esters in the muscles of the three groups whose feeds were supplemented with palm meal (*p* < .05).

**TABLE 7:** Effects of different levels of palm meal feed on the proportion of volatile flavor compounds in Tibetan sheep (%).

Index		ZL‐0	ZL‐15	ZL‐18	ZL‐21	Aroma descriptor
Aldehydes	n‐Nonanal D	0.23 ± 0.02^b^	0.41 ± 0.01^a^	0.26 ± 0.03^b^	0.41 ± 0.06^a^	Herbal, nettle, plastic, soap, citric
	n‐Nonanal M	1.47 ± 0.16^b^	2.78 ± 0.10^a^	1.66 ± 0.21^b^	2.29 ± 0.24^a^	
	Octanal M	1.59 ± 0.08^c^	3.13 ± 0.00^a^	1.93 ± 0.17^bc^	2.14 ± 0.22^b^	Geranium, floral, citrus, lemon
	Octanal D	0.31 ± 0.02^b^	0.62 ± 0.04^a^	0.40 ± 0.08^b^	0.42 ± 0.03^b^	
	Heptanal D	0.34 ± 0.02^c^	0.76 ± 0.03^a^	0.54 ± 0.13^b^	0.35 ± 0.01^c^	Green, fruity, potatoes, rancid
	Heptanal M	1.88 ± 0.07^c^	3.08 ± 0.07^a^	2.70 ± 0.21^b^	2.23 ± 0.16^c^	Burnt; fatty; citrus; nut; sweet
	Hexanal D	1.75 ± 0.12^b^	2.92 ± 0.20^a^	2.83 ± 0.38^a^	2.09 ± 0.35^b^	Grass, green; tallow; fatty
	Hexanal M	2.68 ± 0.21^b^	3.66 ± 0.30^a^	3.68 ± 0.30^a^	3.49 ± 0.47^ab^	
Total		18.31 ± 0.77^c^	25.83 ± 0.63^a^	22.23 ± 0.97^b^	22.47 ± 2.34^ab^	
Alcohols	Isopropanol D	0.16 ± 0.01^a^	0.16 ± 0.02^a^	0.09 ± 0.00^b^	0.12 ± 0.02^b^	Bitterness, rubber, plastic
	Isopropanol M	0.63 ± 0.01^ab^	0.81 ± 0.12^a^	0.62 ± 0.04^b^	0.75 ± 0.06^ab^	
	1‐Butanol D	0.75 ± 0.24^b^	0.44 ± 0.06^b^	1.79 ± 0.03^a^	0.67 ± 0.08^b^	Medicine, fruity, alcohol
	1‐Pentanol M	0.21 ± 0.02^ab^	0.27 ± 0.03^ab^	0.31 ± 0.06^a^	0.18 ± 0.01^b^	Sweety, herbal, oily, nutty
	2‐Ethoxyethanol	0.17 ± 0.02^b^	0.23 ± 0.03^ab^	0.28 ± 0.04^a^	0.29 ± 0.04^a^	Fruity
Total		6.65 ± 0.30	7.80 ± 1.62	8.77 ± 0.44	8.58 ± 0.84	
ketone	3‐Hydroxy‐2‐butanone M	25.24 ± 1.53^a^	23.22 ± 2.26^ab^	18.95 ± 0.55^b^	23.45 ± 1.89^a^	Creamy, dairy, sweety, buttery
	3‐Hydroxy‐2‐butanone D	17.24 ± 1.25^a^	7.43 ± 1.47^b^	10.85 ± 4.48^ab^	13.59 ± 2.92^ab^	
	2‐Butanone T	1.10 ± 0.13^ab^	1.26 ± 0.07^a^	1.00 ± 0.22^ab^	0.83 ± 0.04^b^	Acetone‐like, fruity, camphor
	2‐Propanone	13.39 ± 0.50^c^	21.23 ± 0.76^a^	22.00 ± 1.33^a^	17.00 ± 0.26^b^	Nutty, Bitter
	2‐Pentanone	1.84 ± 0.29^b^	1.47 ± 0.10^c^	3.17 ± 0.23^a^	2.04 ± 0.20^b^	Fruity, sweet
Total		63.94 ± 0.19	60.29 ± 2.55	61.36 ± 1.48	62.01 ± 3.98	
Ester	Methyl acetate	5.40 ± 0.27^a^	0.64 ± 0.05^c^	1.89 ± 0.08^b^	0.88 ± 0.26^c^	Ether, sweety, fruity
	Allyl isothiocyanate	0.16 ± 0.03^b^	0.24 ± 0.03^b^	0.32 ± 0.02^ab^	0.48 ± 0.11^a^	Fruity, ripe, tropical
Total		6.67 ± 0.24^a^	1.97 ± 0.26^c^	3.48 ± 0.05^b^	2.76 ± 0.56^bc^	

*Note*: ZL‐0: PKM was added to feed at 0%, ZL‐15: PKM was added to feed at 15%, ZL‐18: PKM was added to feed at 18%, and ZL‐21: PKM was added to feed at 21%.

Based on these results, it seemed that PKM supplementation influenced the nutritional composition, volatile components, and muscle quality of Tibetan sheep.

### 3.5 The effects of PKM on the metabolome of Tibetan sheep meat

**FIGURE 2:**
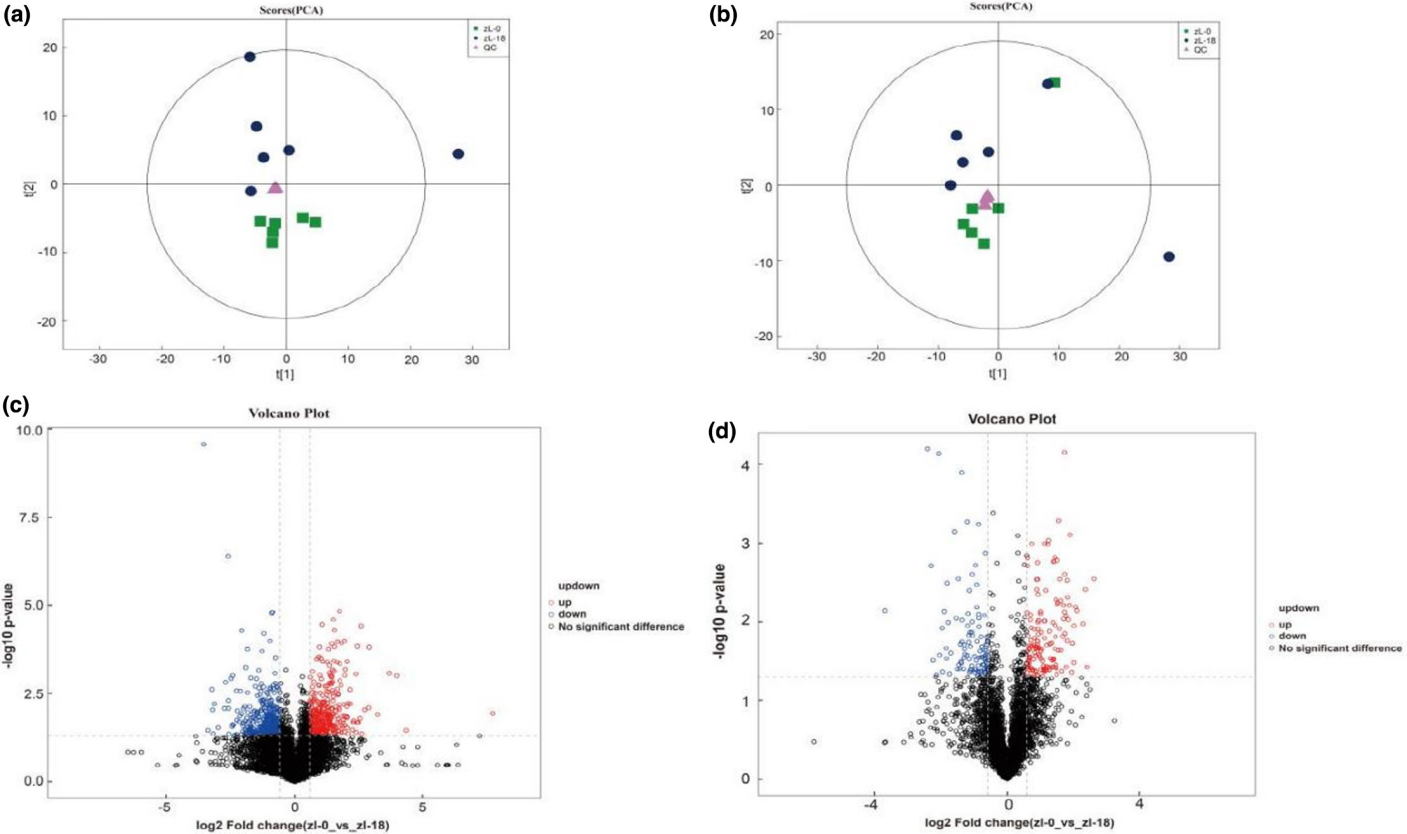
Metabolite profiling analysis of lumborum. Scatter plots of the PCA model based on all identified metabolite features of muscle samples from the fetuses [(a) positive mode; (b) negative mode], including the QC samples. The volcano plot of the comparison between ZL‐0 and ZL‐18 groups (c, positive mode, d, negative mode).

Preliminary phenotypic characterization, AA, FA, and volatile flavor components in Tibetan sheep samples revealed that the addition of 18% palm meal (ZL‐18) to the feed showed optimum effects. Thus, to better understand the changes in muscle metabolites after the addition of palm meal, two groups, namely ZL‐0 and ZL‐18, were selected to compare their metabolite profiles using metabolomic analysis. Figure 2 shows the PCA score plots and the volcano plots for the two groups, along with the QC samples in positive and negative ion modes. Overall, the muscle metabolites were well separated between the two groups. Figure S1 further shows the OPLS‐DA model obtained by orthogonal partial least squares discriminant analysis, while Table S3 lists the model evaluation parameters (R^2^Y, Q^2^) obtained by a sevenfold cross‐validation. It was noted that the OPLS‐DA model, for which stability was good, could distinguish two groups of samples. Indeed, 70 differential metabolites were identified between the two based on OPLS‐DA, with VIP > 1 and *p* value = .05 selected as thresholds (37 in positive ion mode and 23 in negative ion mode). The ZL‐18 group was also found to have nine up‐regulated metabolites and 14 down‐regulated ones compared with the ZL‐0 group in the negative ion detection mode (Table 8). Information on differential metabolites in positive ion assay mode is provided in the Supplemental Material (Table S2).

**TABLE 8:** The detailed results of differential metabolites in the longissimus lumborum in the positive ion detection mode (OPLS‐DA VIP > 1 and *p* < .05).

Name	Adduct	m/z	Rt(s)	VIP	FC	Variation	*p*‐value
Dl‐a‐hydroxybutyric acid	[M‐H]‐	103.04	284.36	1.98	1.52	↑	.00
N‐(2,4‐dinitrophenyl)‐l‐leucine	[M‐H‐CH_4_O_4_]‐	216.06	34.24	1.52	5.20	↑	.00
Chlorophene	[M‐H]‐	217.05	34.6951	2.91	3.30	↑	.01
Sarcosine	[M‐H]‐	88.04	381.80	2.57	1.08	↑	.01
Cis‐aconitate	[M‐H‐CH_2_O_3_]‐	111.01	403.439	3.17	2.18	↑	.01
DL‐proline	[M‐H]‐	114.06	368.02	1.91	1.35	↑	.01
Met‐Phe	[M‐H]‐	295.11	264.24	1.47	2.40	↑	.01
Uridine	[M‐H]‐	243.06	294.46	2.33	0.74	↓	.01
Dopamine	[M‐H]‐	152.00	270.28	2.87	0.45	↓	.01
Erucic acid	[M‐H]‐	337.31	67.57	1.48	0.53	↓	.01
L‐Ascorbic acid	(M + Na‐2H)‐	197.01	224.95	2.08	1.67	↑	.02
Dulcitol	[M‐H]‐	181.07	363.32	2.83	1.83	↑	.02
1,4‐Butynediol	[M‐H]‐	85.03	385.24	1.39	1.51	↑	.02
Sm d34:1	[M + CH_3_COOH‐H]‐	761.58	274.05	1.24	1.81	↑	.03
Succinate	[M‐H]‐	117.02	368.52	4.68	0.50	↓	.03
1‐Palmitoyl‐2‐hydroxy‐sn‐glycero‐3‐Phospho‐(1′‐rac‐glycerol)	[M‐H]‐	483.27	68.17	1.63	0.83	↓	.03
N‐acetylputrescine	[M‐H]‐	129.09	113.10	1.40	0.75	↓	.04
5‐Methylbenzotriazole	[M‐H]‐	132.05	107.47	1.76	5.38	↑	.04
Isocitric acid	[M‐H‐H_2_O]‐	173.01	386.08	1.70	1.49	↑	.04
Uracil	[M‐H]‐	111.02	220.15	2.37	0.47	↓	.04
11‐Dehydrothromboxane b2	[M‐H]‐	367.22	67.57	1.79	0.46	↓	.04
4,4′‐Thiodiphenol	[M‐H]‐	217.02	39.98	1.19	0.30	↓	.05
Pantothenic acid	[M‐H]‐	218.10	330.97	2.06	1.27	↑	.05

**FIGURE 3:**
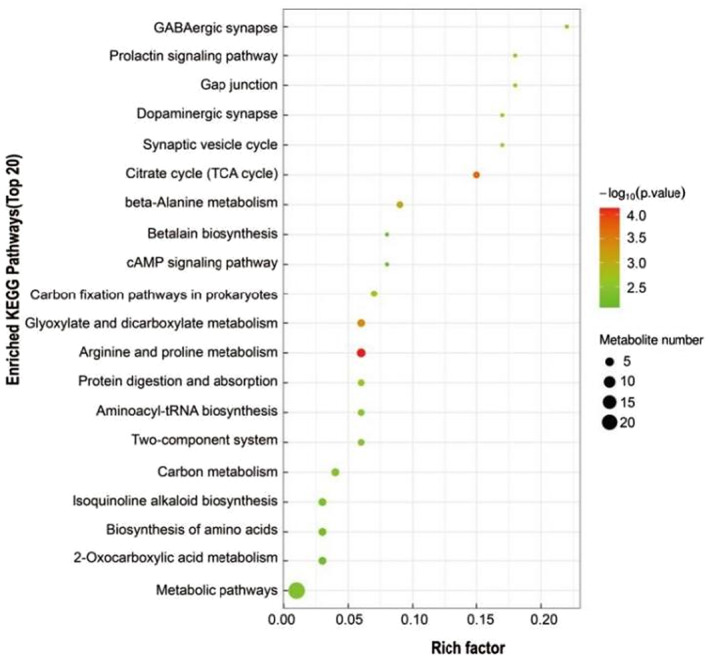
Top 20 enriched KEGG pathways of the comparison between ZL‐0 and ZL‐18 groups. In the bubble diagram, each bubble represents a metabolic pathway. The larger the bubble, the greater the impact factor; and the darker the bubble, the more significant the degree of enrichment.

**FIGURE 4:**
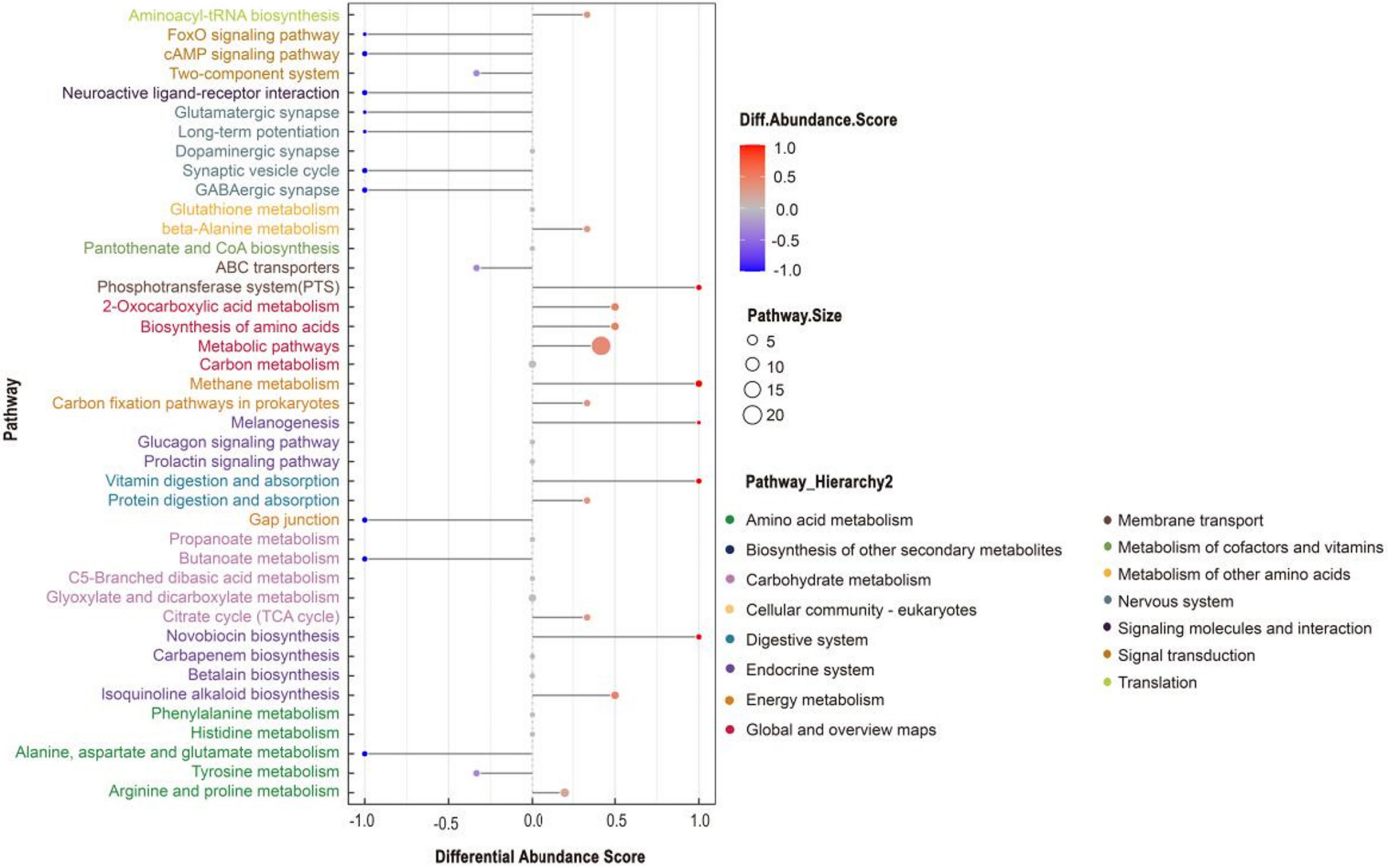
A differential abundance score map of differential metabolic pathways. The differential abundance score captures the average, gross changes for all metabolites in a pathway. A score of 1 indicates all measured metabolites in a pathway decrease, and −1 indicates all measured metabolites in a pathway increase in the ZL‐18 group.

Based on KEGG pathway enrichment analysis, Figure 3 shows the top 20 metabolic pathways affected by the addition of PKM, with most being involved in carbohydrate metabolism and amino acid metabolism. Based on differential abundance analysis (DA) plots, at least 41 metabolic pathways and 25 metabolites were identified (*p* < .05), with 10 being up‐regulated (<−0.5 DA score, blue) and 8 being down‐regulated (<0.5 DA score) in the ZL‐18 group (Figure 4). Of these, the main up‐regulated ones included butanoate metabolism as well as alanine, aspartate, and glutamate metabolism. On the other hand, phosphotransferase systems (PTS), 2‐oxocarboxylic acid metabolism, biosynthesis of amino acids, novobiocin biosynthesis, and isoquinoline alkaloid biosynthesis were down‐regulated.

It was also found that DL‐Glu levels were higher in the ZL‐18 group, while DL‐tyr, DL‐pro, Sar, DL‐arg, and N‐(2,4‐dinitrophenyl)‐l‐leucine levels were lower (VIP > 1.00, *p* < .05) (Table 7 and Table S2). These results suggest that the PKM feed led to changes in some of the LL's amino acids, thereby affecting the flavor of the meat. As shown in Figure 7, 13 metabolites were involved in the TCA cycle pathway as well as in the synthesis and degradation of some amino acids. In this pathway, compared with the ZL‐0 group, succinate, N‐acetylputrescine, uridine, dopamine, uracil, and erucic acid were up‐regulated and Dl‐a‐hydroxybutyric acid, sarcosine, Cis‐aconitate, DL‐proline, isocitric acid, L‐ascorbic acid, and pantothenic acid were down‐regulated in the ZL‐18 group.

### 3.6 The effects of PKM on the rumen bacterial communities of Tibetan sheep meat

**FIGURE 5:**
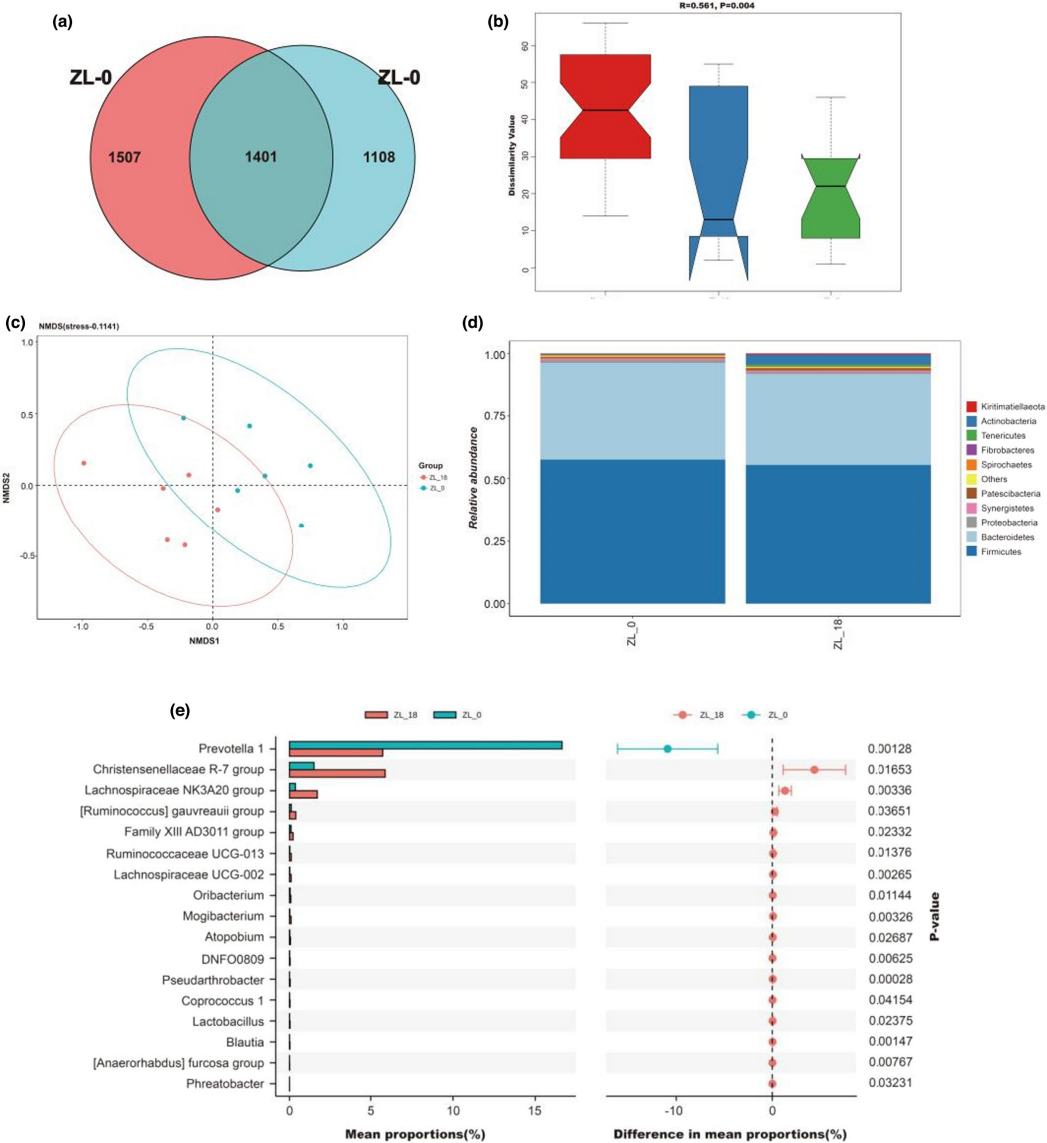
Venn diagram illustrating the overlap of microbial OTUs between the two groups (a). Anosim (analysis of similarities) based on Bray–Curtis distances between the three groups (b). NMDS of taxonomical classifications of bacterial communities (c). Relative abundance of bacteria community proportions at the phylum (d). The proportion of abundance of different species in ZL‐0 and ZL‐18 is shown in the middle as the ratio of differences within the 95% confidence interval. The rightmost value is the *p*‐value, and a *p*‐value <.05 indicates a significant difference (e).

As previously mentioned, there were significant differences in metabolite levels between the two groups of samples, and it is not unlikely that changes in rumen microbes impacted muscle metabolism. For this purpose, 16S rDNA sequencing was used to characterize the rumen microbiota of both ZL‐0 and ZL‐18 groups in order to determine the relationship between microbiota and various metabolites. A total of 4016 OTUs were identified, with 1507 and 1108 being specific to ZL‐0 and ZL‐18, respectively (Figure 5a). Based on the Bray–Curtis algorithm, Anosim analysis was performed, with the results, shown in Figure 5b and Table S2, indicating significant differences between the two groups. For ecological research, Non‐Metric Multi‐Dimensional Scaling (NMDS) statistics can be used. In a multi‐dimensional space, the species information is represented in the form of points according to the species information contained in the sample. It can reflect differences between and within groups of samples based on the distance between points. As shown in Figure 5c, the rumen flora for the two groups of Tibetan sheep were clearly distinct, hence highlighting differences between the two. In order to further clarify the microbial communities of groups ZL‐0 and ZL‐18, the OTUs were classified at the phylum and genus levels.

Following taxonomic classification, Firmicutes and Bacteroidetes were found to dominate the two groups. Indeed, they are important phyla of Tibetan sheep's rumen (Figure 5d). The abundance of species between the two groups of samples was further compared using Welch's t‐test analysis. As shown in Figure 5e, for the ZL‐18 group, the relative abundance of *Christensenellaceae R‐7 group, [Ruminococcus] gauvreauii group, Family XIII AD3011 group, Ruminococcaceae UCG‐013*, and *Lachnospiraceae UCG‐002* was significantly higher than that in the zL‐0 group (*p* < .05), indicating that these bacteria were found in greater numbers in Tibetan sheep's rumens after PKM was added to their feed. However, the relative abundance of *Prevotella 1* in ZL‐0 group was higher (*p* < .05).

### 3.7 Correlation analysis

**FIGURE 6:**
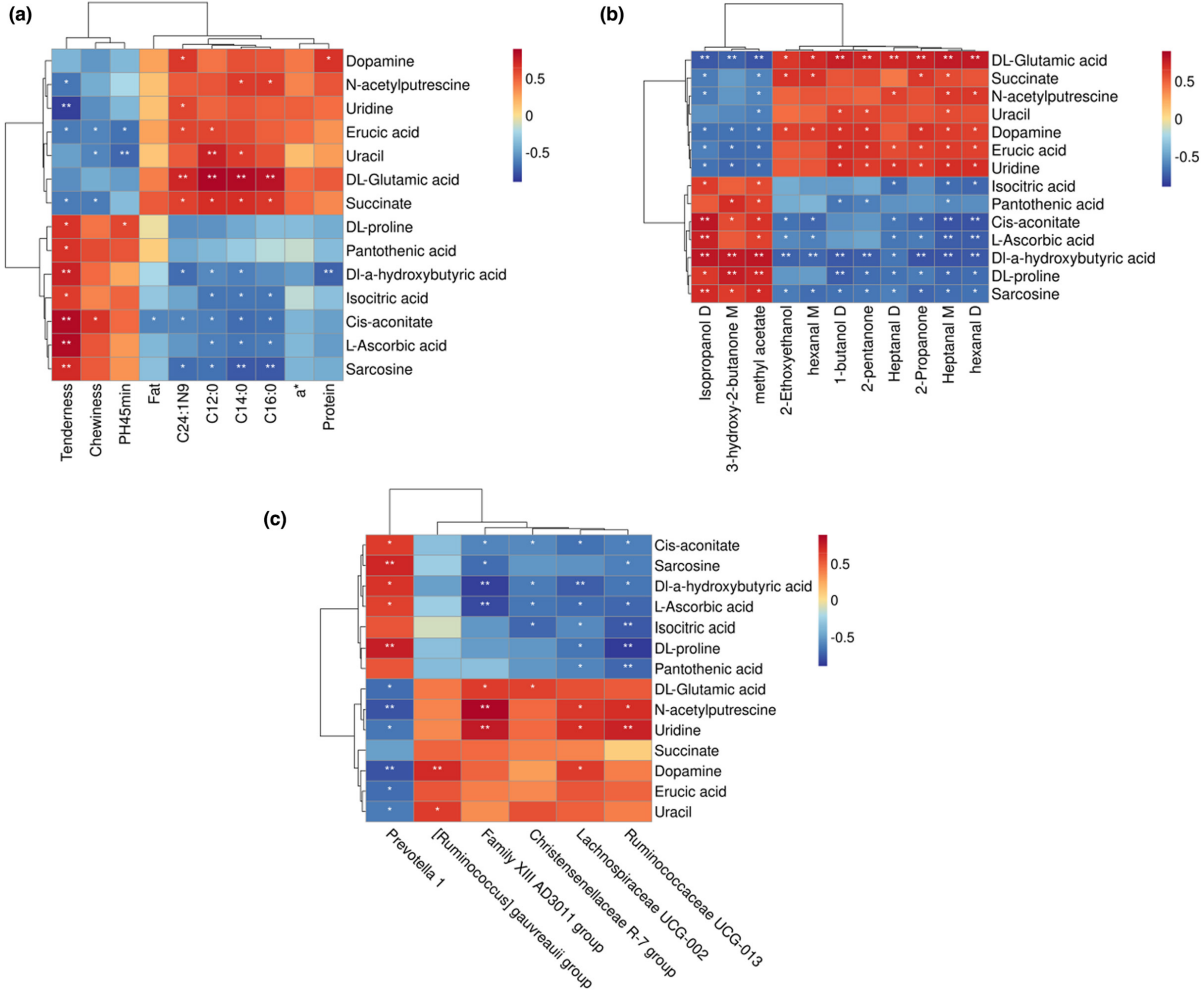
Pearson's correlations between key DFMs and meat quality (a), volatile flavor components (b), and different rumen microbiomes (c). The significant correlations (*r* > .52 or *r* < −.52, *p* < .01) were shown in the correlation heatmaps. The color intensity and circle size are proportional to the correlation values.

We ventured to speculate that perhaps by changing the feed composition we could change the rumen colony composition and thus the muscle metabolism, thus controlling the quality of Tibetan sheep meat in a targeted manner. In this context, the relationship among four different species, different metabolites, meat quality, and volatile flavor substances in Tibetan sheep samples was explored. As seen on the thermogram (Figure 6a), muscle tenderness (indicated by shear force), chewiness, and pH_45 min_ were positively correlated with Dl‐a‐hydroxybutyric acid, sarcosine, cis‐aconitate, DL‐proline, isocitric acid, L‐ascorbic acid, and pantothenic acid, while the four fatty acids (C24:1 N9, C12:0, C14:0, and C16:0) content in LL, the protein levels, and the a* were positively correlated with succinate, N‐acetylputrescine, uridine, dopamine, uracil, erucic acid, and DL‐glutamic acid.

In the correlation analysis of muscle metabolites with volatile flavor substances (Figure 6b), isopropanol D, 3‐hydroxy‐2‐butanone M, and methyl acetate correlated positively with Dl‐a‐hydroxybutyric acid, sarcosine, cis‐aconitate, DL‐proline isocitric acid, and L‐ascorbic acid. Pantothenic acid was positively correlated with four types of aldehydes (heptanal D, heptanal M, hexanal D, and hexanal M), and 1‐butanol D, 2‐ethoxyethanol, 2‐propanone, and 2‐pentanone were positively correlated with DL‐glutamic acid, succinate, N‐acetylputrescine, uridine, dopamine, uracil, and erucic acid.

There was also a close relationship between the formation of metabolites and microbiota. As shown in Figure 6c, *Prevotella 1* was positively correlated with Dl‐a‐hydroxybutyric acid, sarcosine, cis‐aconitate, DL‐proline, isocitric acid, L‐ascorbic acid, and pantothenic acid, while DL‐ glutamic acid, succinate, N‐acetylputrescine, uridine, dopamine, uracil, and erucic acid were negatively correlated. Similarly, *Christensenellaceae R‐7 group, [Ruminococcus] gauvreauii group, Family XIII AD3011 group, Lachnospiraceae UCG‐002*, and *Ruminococcaceae UCG‐013* were correlated with DL‐glutamic acid, succinic acid, N‐acetylputrescine, uridine, dopamine, uracil, and erucic acid, but was negatively correlated with Dl‐a‐hydroxybutyric acid, sarcosine, cis‐aconitate, DL‐proline, isocitric acid, L‐ascorbic acid, and pantothenic acid. In addition, the correlation between muscle phenotypic characteristics and rumen microorganisms is shown above the heatmap (Figure S2A). The correlation between muscle volatile flavor substances and rumen microorganisms is also shown in the heatmap (Figure S2B).

## 4 DISCUSSION

PKM contains valuable dietary sources of protein, amino acids, energy, and fiber and it has grown to become a proven feed ingredient for poultry and pig diets, especially since it can be applied at low levels with ruminant feeds (Ng, 2003). If it is added to Tibetan sheep feed, it can effectively reduce feed costs. However, the addition of PKM and its effects on the quality of Tibetan sheep are yet to be extensively studied, and as such, the mechanisms underlying meat quality changes remain unclear. In this context, the changes that occurred in meat quality, muscle metabolism, and rumen flora were explored after providing palm meal feed to Tibetan sheep in order to assess possible beneficial or detrimental effects of PKM on the meat quality of Tibetan sheep.

**FIGURE 7:**
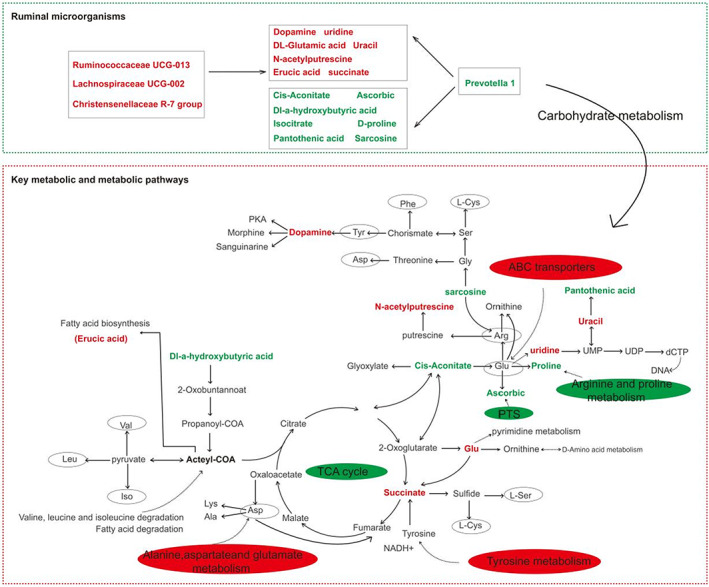
Scheme of the primary metabolic pathway and relative metabolite contents in ZL‐18 and ZL‐0. The metabolic pathway was adopted from the KEGG database (KEGG, http://www.genome.jp/kegg). Red represents the up‐regulated metabolites, key flora, and metabolic pathways in the ZL‐18 group; and green represents the down‐regulated metabolites, key flora, and metabolic pathways in the ZL‐18 group. The gray circles are the downstream amino acids in some metabolic pathways.

In this study, the addition of PKM had no significant effect on the carcass shape of Tibetan sheep, consistent with the results of Jang et al (Jang et al., 2020) and Silva et al. (2020) (Ribeiro et al., 2018). Some changes in Tibetan sheep meat quality occurred after providing PKM diets. For example, muscle pH and a* values decreased, with the most obvious being in terms of muscle tenderness which improved in all three groups that were fed PKM diets. Higher pH can lead to darker muscle color, firmer and drier meat, shorter shelf life, as well as poorer appearance of meat. Altogether, these features affect the quality and safety of meat (Kirkpatrick et al., 2022; Newton & Gill, 1981). The hypoxic state of muscle cells in postmortem muscles prevented the production of large amounts of ATP through the citric acid cycle and oxidative phosphorylation, thus allowing glycolysis to gradually dominate the ATP production process, and leading to lactate accumulation and pH decrease (Warner et al., 2021). However, after the addition of palm meal to the feed, Dl‐a‐hydroxybutyric acid, isocitric acid, and cis‐aconitate were down‐regulated, further blocking the citric acid cycle pathway. In fact, this could have been the reason for the lower pH_45 min_ in the ZL‐18 group as succinate dehydrogenase during the TCA cycle is a potential meat tenderizing process (Ouali et al., 2013). Succinate up‐regulation in the ZL‐18 group may be accompanied by an enrichment of succinate dehydrogenase, which would then explain the reduction in muscle shear in Tibetan sheep after palm meal feeding. These results indicated that tender meat was obtained at low pH, since it causes the contraction of muscles, hardening of texture, and the production of pale meat, especially when the pH drops rapidly (Bowker et al., 1999; Hammelman et al., 2003). The initial pH_45 min_ of the ZL‐15 group was 6.01, which was higher than the other three groups. However, after 24 h, its pH was basically the same as for the other groups, and this could explain the values of a* and shear force for the ZL‐15 group relative to the other groups. After slaughtering the animals, myofilaments contract and relax continuously. At the same time, glycolysis decreases glycogen levels and depletes ATP, leading to myofilament contraction stiffness (Hopkins & Ertbjerg, 2023). In addition, pyruvate is converted by the action of pyruvate dehydrogenase complex to acetyl coenzyme A, which then enters the TCA cycle (Krebs & Johnson, 1980). This cycle was down‐regulated after feeding PKM diets, probably due to the fact that feeding PKM diets can also inhibit muscle glycolysis in Tibetan sheep at later stages, thus delaying the pH drop, the myofilament contraction, and the higher muscle tenderness (Figure 7). Figure S2A and Figure 7 showed that shear force was strongly and positively correlated with *Lachnospiraceae UCG‐002, Ruminococcaceae UCG‐013*, and *Family XIII AD3011 group*, although these three bacteria were also negatively correlated with isocitric acid, a key substance involved in the TCA cycle, ci‐aconitate, and Dl‐a‐hydroxybutyric. The levels of these three metabolites were also reduced in the Tibetan sheep group fed PKM diets. The *Lachnospiraceae UCG‐002, Ruminococcaceae UCG‐013*, and *Family XIII AD3011 groups* influenced the deposition of metabolites to muscle through carbohydrate metabolism (Bach et al., 2019; Guo et al., 2018). PTS imports and phosphorylates carbon sources (e.g., glucose) and converts phosphoenolpyruvate to pyruvate (Figure 6). The pyruvate then inhibits glucose phosphorylation and cAMP production to further inhibit glycolysis (Lee et al., 2005; Zhang, Han, Hou, Raza, Gui, et al., 2022). Additionally, it inhibited the phosphorylation of APK (camp‐dependent protein kinase) signaling pathway, resulting in a decrease in glycogen metabolism (McCloskey et al., 2018). It was also speculated that up‐regulation of phosphotransferase system in ZL‐18 group decreased the level of cAMP, which also further regulated the pH decrease and improved LL tenderness of ZL‐18. The mechanisms through which PKM addition affect phosphotransferase system of sheep muscle cell still require further study.

Living organisms require protein for normal development since it is a major source of essential amino acids (Borba et al., 2022). It was found that replacing a certain percentage of commercial feed with fermented rice bran resulted in protein deposition in chicken meat (Al‐Arif et al., 2020). Similarly, feeding diets with a protein content of 130 g/kg DM resulted in more proteins in goat meat muscle (Atti et al., 2004), while in this study, effective protein deposition in muscle was also found after feeding Tibetan goats with PKM instead of soybean meal. These findings suggested that protein in feed affects protein deposition in muscle. Studies have shown that activating the AKT/mammalian rapamycin (mTOR) pathway and increasing the phosphorylation activity of S6K1 can regulate protein deposition in muscle (Liang et al., 2022). Its protein complexes named mTOR_C1_ can then promote cell growth by inhibiting proteolytic metabolism, while amino acids stimulate the conversion of the regulator to an active nucleotide‐binding state, thereby allowing it to bind raptor and recruit mTOR_C1_ to the lysosomal surface (Saxton & Sabatini, 2017). It is speculated that alanine, aspartate, glutamate, and tyrosine metabolism pathways are up‐regulated in the ZL‐18 group, thereby activating mTORC1 and allowing protein deposition (Figure 3).

The saturated fatty acids of pork tenderloin Increased when the level of PKM in the diet increased (An et al., 2017). Interestingly, similar results were obtained in Tibetan sheep LL. C12:0, a specific type of fatty acid found in saturated fatty foods, produces impressive immune‐enhancing effects in the body, and it ranks first among more than 30 fatty acids for its antimicrobial properties (Hoa et al., 2022; Kumar et al., 2020). In contrast, C14:0 is used as a flavor component in the food industry (Burdock & Carabin, 2007) and C24:1n9 is a monounsaturated fatty acids (MUFA), which is an “advanced nutrient” necessary for nerve cells, especially brain cells, optic nerve cells, and peripheral nerve growth. It is also important for the redevelopment and maintenance of physiological functions, and it needs to be taken in vitro due to the body's inability to produce it. These fatty acids correlated with muscle metabolites such as sarcosine, cis‐aconitate, and DL‐glutamic acid, and could have been enriched by carbohydrate metabolism after feeding palm meal.

The volatile flavor of Tibetan sheep meat can largely determine its consumption in the market. PKM feed increased the proportion of aldehydes but decreased the proportion of esters in Tibetan sheep LL (Table 6). Of the different types of aldehydes produced during muscle oxidation, nonanal, hexanal, heptanal, and octanal are key volatile flavor compounds due to their low perception threshold as well as their ability to give the muscles a fresh taste of grasses and legumes (Huang et al., 2022). However, octanal and nonanal may also impart off‐flavors and sourness (Huang et al., 2022), and the meat may give off a rotten smell when the levels are exorbitantly high. These aldehydes increased in content after the addition of 15% and 18% PKM, but the levels of octanal and nonanal were higher in the ZL‐15 group. Isopropyl alcohol has a bitter and plastic taste, and its proportion decreased after the addition of 18% PKM. 1‐butanol D and 2‐ethoxyethanol also have pleasant odors, such as sweetness and fruitiness (Wang, Li, et al., 2022; Wang, Zhao, et al., 2022), and their proportions increased after the addition of PKM. Finally, ketones have a high threshold, mostly possessing a creamy odor (Huang et al., 2022), and having a positive effect on volatile flavors, while the proportions of 2‐propanone and 2‐pentanone increased in the ZL‐18 group. Unfortunately, methyl acetate also had good flavor for meat, but its percentage decreased after the addition of palm meal. In conclusion, ZL‐18 showed more advantages in flavor overall.

Volatile flavor substances are mainly composed of aldehydes, alcohols, and ketones produced by lipid oxidation (Liu et al., 2020). In the aldehydes, hexanal and heptanal are produced by the oxidation of linoleic and arachidonic acids, respectively. On the other hand, octanal and nonanal are produced by oleic acid oxidation. The production of pentanol in alcohols is formed by the oxidation of polyunsaturated fatty acids (Huang et al., 2022). Meanwhile, the biosynthesis of pentanol and 1‐butanol D is initiated by acetyl coenzyme A (Ko et al., 2022; Nitta et al., 2019), and 3‐hydroxy‐2‐butanone decreases its own proportion by oxidative decarboxylation that destroys alkyl radicals and by the combination of propyl radicals with those generated by fatty acid oxidation to produce furans (Wang et al., 2021). This also suggests that VOCs can be further associated with some metabolic pathways in muscle. Previously it was found that the composition of fatty acids is related to carbohydrate metabolism, and here, it was speculated that there could also be a link between VOCs and carbohydrate metabolism. In the correlation analysis (Figure 5b), VOCs were strongly correlated with the intermediate metabolites' succinate, erucic acid, and dopamine, which are involved in the TCA cycle. These results provided additional verification that the deposition of VOCs is related to the carbohydrate metabolic pathway and that pyruvate is a key flavor intermediate that is produced through the carbohydrate metabolic pathway (TCA cycle) (Chen et al., 2017). In addition, most of the volatile compounds with odor activity are plant based, with their accumulation in animal tissues (e.g., muscle) being also associated at the intramuscular fat deposition (Vasta & Priolo, 2006). In addition, studies have shown that amino acids are precursors to VOCs, such as the degradation of alanine to form hexanal as well as the conversion of aspartic acid to oxaloacetic acid by transaminases. These reactions also yield n‐butanol, 2‐butanone, and other ketones. Isovaleraldehyde and isobutyraldehyde can be produced from leucine and isoleucine, and further reactions can be conducted to produce alcohols and acids from these aldehydes. This suggests that the synthesis of VOCs may also be related to alanine, aspartate, glutamate, tyrosine, arginine, and proline metabolic pathways.

Diet plays an important role in shaping the rumen microbiome, with one previous study showing that the rumen's metabolite levels were influenced by a high‐energy diet. This led to an increase in *Quinella*, *Ruminococcus*, and *UCG‐001*, all of which participate in carbohydrate metabolism of Tibetan sheep (Zhang, Han, Hou, Raza, Gui, et al., 2022). In addition, some studies have found that the addition of fiber‐rich substances in feed can promote the proliferation of some fiber‐dissolving bacteria. For example, the *Ruminococcaceae* represents a common family of fiber‐degrading bacteria that hydrolyzes complex carbohydrates in ruminants (Liu et al., 2020; Song et al., 2018). Similar to the *Ruminococcaceae family*, *Christensenellaceae* are considered to be fiber‐degrading bacteria, with highly abundant populations of these bacteria found in the rumen of sows fed with sugar beet pulp, a type of fiber (Shang et al., 2021). In addition, *Lachnospiraceae* are associated with carbohydrate metabolism and vitamin B12 synthesis in the rumen (Reeves et al., 2012; Vacca et al., 2020). This study showed that the addition of PKM to the diet promoted the proliferation of *Christensenellaceae R‐7 group*, *[Ruminococcus] gauvreauii group*, *Family XIII AD3011 group, Ruminococcaceae UCG‐013*, and *Lachnospiraceae UCG‐002* at the genus level and prevented the proliferation of *Prevotella 1*. Therefore, it is speculated that the reason for these changes may be related to the fact that PKM itself contains more crude fiber (Azizi et al., 2021). Similarly, cellulolytic bacteria are capable of hydrolyzing polysaccharides into fermentable sugars from feed substrates, thus promoting rumen metabolism (Froidurot & Julliand, 2022). Lowering dietary fiber, therefore, results in lower muscle pH and improved fatty acid distribution (Han et al., 2020; Santos‐Silva et al., 2020), which is consistent with the current findings. Based on the correlation analysis, these up‐regulated bacteria were positively correlated with protein, fat, C12:0, C14:0, C16:0, C24:1 N9, and flavor substances such as heptanal, hexanal, and 2‐ethoxyethanol, On the other hand, they were negatively correlated with pH, shear, chewiness, and flavor substances such as isopropanol D. This may prove that the carbohydrate metabolic pathway is an important means by which ruminates alters the meat quality of Tibetan sheep. Most studies have confirmed that PKM as a component in feeds and in the right amounts does not have any harmful effects on animal growth performance and meat quality, and also increases consumer acceptance of sheep meat (Choi et al., 2021). However, further research is needed to determine how PKM feed alters rumen flora composition to affect Tibetan sheep meat quality.

## 5 CONCLUSION

Numerous studies have shown that PKM is a high‐quality protein source in ruminant feeds (crude protein, 12%–21%). In this work, the Tibetan sheep meat of ZL‐18 group had better eating quality, while the 18% PKM addition promoted the protein and fat deposition in the muscle. On the other hand, PKM feeds had little effect on AA levels in LL, but for FA, PKM feeds caused an increase in the levels of saturated fatty acids in LL. Furthermore, adding PKM to the feed resulted in an increase in muscle aldehydes and a decrease in muscle esters. In muscle metabolism, mainly based on carbohydrate metabolism, LL pH, tenderness, and flavor were altered by up‐regulating the PTS, down‐regulating the TCA cycle pathway, and having both up‐ and down‐regulating amino acid pathways. The rumen bacteria, including key genera such as *Ruminococcaceae UCG‐013, Christensenellaceae R‐7 group*, and *Lachnospiraceae UCG‐002*, differed significantly between ZL‐0 and ZL‐18. The correlations of rumen bacteria and meat quality, and metabolism indicated the addition of PKM may improve the quality and flavor of Tibetan sheep meat by affecting microorganisms in the rumen and muscle metabolism.

## AUTHOR CONTRIBUTIONS

Conceptualization, Ying Ma, Lijuan Han, Xue Zhang, Shengzhen Hou, Linsheng Gui, Shengnan Sun, Zhenzhen Yuan, Zhiyou Wang, and Baochun Yang; Writing—original draft, Ying Ma and Sayed Haidar Abbas Raza; Writing—review & editing; Lijuan Han, Data curation, Ying Ma, Lijuan Han, and Zhenzhen Yuan; Formal analysis, Xue Zhang, Linsheng Gui, and Baochun Yang; Investigation, Zhiyou Wang, Shengnan Sun, and Shengzhen Hou; Methodology, Ying Ma, Lijuan Han, and Baochun Yang; Project administration, Lijuan Han and Shengzhen Hou; Resources and Software, Baochun Yang and Linsheng Gui; Supervision, Lijuan Han and Shengzhen Hou; Validation, Zhiyou Wang and Shengnan Sun; Formal analysis, Investigation, Methodology Validation, Writing—review & editing, Mohamed M. Hassan, Ruqaih S Alghsham, Waleed Al Abdulmonem, and Samia S. Alkhalil. All authors have read and agreed to the published version of the manuscript.

## FUNDING INFORMATION

The current work was funded by the Construction of Standardized Production System for improving quality and efficiency of Tibetan Sheep industry (2022‐NK‐169).

## CONFLICT OF INTEREST STATEMENT

There is no conflict of interest in the present study. All authors consent to participate in and publish this article. The authors acknowledge Xingang Lv (Shanghai Applied Protein Technology Co., Ltd., China) for the technical support. The authors are thankful for the support of Qinghai University for the use of laboratory facilities. The authors declare that the research was conducted in the absence of any commercial or financial relationships that could be construed as a potential conflict of interest.

## ETHICS APPROVAL AND CONSENT TO PARTICIPATE

This study was carried out in strict accordance with the animal protection and use guidelines established by the Ministry of Science and Technology of the People's Republic of China. All animal care and handling were approved by the Institutional Animal Care and Use Committee Guidelines of Qinghai University (QUA‐2020‐0709). Moreover, all applicable rules and regulations of the organization and government were followed regarding the ethical use of experimental animals.

## Supporting information


Figure S1.
Figure S2.
Table S1.
Table S2.
Table S3.

## Data Availability

The 16S rDNA sequencing data presented in the study are deposited in the NCBI repository, accession number PRJNA917527.

## References

[fsn33340-bib-0001] Akinyeye, R. O. (2011). Physico‐chemical properties and anti‐nutritional factors of palm fruit products (Elaeis Guineensis Jacq.) from Ekiti state Nigeria. Electronic Journal of Environmental, Agricultural and Food Chemistry (EJEAFChe), 10(5), 2190–2198.

[fsn33340-bib-0002] Al‐Arif, M. A. , Warsito, S. H. , Amin, M. , & Lamid, M. (2020). Substitution of commercial feed with phytase‐fermented rice bran and turmeric flour to increase EPA, DHA, and protein depositions in broiler meat. Biocatalysis and Agricultural Biotechnology, 24, 101535.

[fsn33340-bib-0003] Alshelmani, M. I. , Loh, T. C. , Foo, H. L. , Sazili, A. Q. , & Lau, W. H. (2016). Effect of feeding different levels of palm kernel cake fermented by *Paenibacillus polymyxa* ATCC 842 on nutrient digestibility, intestinal morphology, and gut microflora in broiler chickens. Animal Feed Science and Technology, 216, 216–224.

[fsn33340-bib-0004] An, J. Y. , Yong, H. I. , Kim, S. Y. , Yoo, H. B. , Kim, Y. Y. , & Jo, C. (2017). Quality of frozen pork from pigs fed diets containing palm kernel meal as an alternative to corn meal. Korean Journal for Food Science of Animal Resources, 37(2), 191–199. 28515643 10.5851/kosfa.2017.37.2.191 PMC5434206

[fsn33340-bib-0005] Atti, N. , Rouissi, H. , & Mahouachi, M. (2004). The effect of dietary crude protein level on growth, carcass and meat composition of male goat kids in Tunisia. Small Ruminant Research, 54(1–2), 89–97.

[fsn33340-bib-0006] Azizi, M. N. , Loh, T. C. , Foo, H. L. , & Teik Chung, E. L. (2021). Is palm kernel cake a suitable alternative feed ingredient for poultry? Animals, 11(2), 338. 33572711 10.3390/ani11020338 PMC7911022

[fsn33340-bib-0007] Bach, A. , López‐García, A. , González‐Recio, O. , Elcoso, G. , Fàbregas, F. , Chaucheyras‐Durand, F. , & Castex, M. (2019). Changes in the rumen and colon microbiota and effects of live yeast dietary supplementation during the transition from the dry period to lactation of dairy cows. Journal of Dairy Science, 102(7), 6180–6198. 31056321 10.3168/jds.2018-16105

[fsn33340-bib-0008] Borba, K. K. S. , Gadelha, T. S. , Sant'Ana, A. M. S. , Pacheco, M. T. B. , Pinto, L. S. , Madruga, M. S. , Medeiros, A. N. , Bessa, R. J. B. , Alves, S. P. A. , Magnani, M. , Pimentel, T. C. , & do Egypto Queiroga, R. D. C. (2022). Fatty acids, essential amino acids, minerals and proteins profile in whey from goat cheese: Impacts of raising system. Small Ruminant Research, 217, 106842.

[fsn33340-bib-0009] Bowker, B. C. , Wynveen, E. J. , Grant, A. L. , & Gerrard, D. E. (1999). Effects of electrical stimulation on early postmortem muscle pH and temperature declines in pigs from different genetic lines and halothane genotypes. Meat Science, 53(2), 125–133. 22063089 10.1016/s0309-1740(99)00043-1

[fsn33340-bib-0010] Burdock, G. A. , & Carabin, I. G. (2007). Safety assessment of myristic acid as a food ingredient. Food and Chemical Toxicology, 45(4), 517–529. 17141389 10.1016/j.fct.2006.10.009

[fsn33340-bib-0011] Caporaso, J. G. , Lauber, C. L. , Walters, W. A. , Berg‐Lyons, D. , Lozupone, C. A. , Turnbaugh, P. J. , Fierer, N. , & Knight, R. (2011). Global patterns of 16S rRNA diversity at a depth of millions of sequences per sample. Proceedings of the National Academy of Sciences of the United States of America, 108(11SUPPL), 4516–4522. 20534432 10.1073/pnas.1000080107 PMC3063599

[fsn33340-bib-0012] Chen, C. , Zhao, S. , Hao, G. , Yu, H. , Tian, H. , & Zhao, G. (2017). Role of lactic acid bacteria on the yogurt flavour: A review. International Journal of Food Properties, 20(sup1), S316–S330.

[fsn33340-bib-0013] Chen, Q. , Zhang, W. , Cai, J. , Ni, Y. , Xiao, L. , & Zhang, J. (2022). Transcriptome analysis in comparing carcass and meat quality traits of Jiaxing black pig and Duroc × Duroc × Berkshire × Jiaxing black pig crosses. Gene, 808, 145978. 10.1016/j.gene.2021.145978 34592352

[fsn33340-bib-0014] Choi, W. J. , Kim, J. H. , Kim, H. W. , Kim, K. E. , & Kil, D. Y. (2021). Effects of dietary palm kernel meal and β‐xylanase on productive performance, fatty liver incidence, and excreta characteristics in laying hens. Journal of Animal Science and Technology, 63(6), 1275–1285. 34957443 10.5187/jast.2021.e111 PMC8672254

[fsn33340-bib-0015] Corley, R. H. V. (2009). How much palm oil do we need? Environmental Science & Policy, 12(2), 134–139.

[fsn33340-bib-0019] DeSantis, T. Z., Jr. , Hugenholtz, P. , Keller, K. , Brodie, E. L. , Larsen, N. , Piceno, Y. M. , Phan, R. , & Andersen, G. L. (2006). NAST: A multiple sequence alignment server for comparative analysis of 16S rRNA genes. Nucleic Acids Research, 34, W394–W399. 10.1093/nar/gkl244 16845035 PMC1538769

[fsn33340-bib-0020] Devapriya, A. , Sejian, V. , Ruban, W. , Devaraj, C. , Spandan, P. V. , Silpa, M. V. , Reshma Nair, M. R. , Nameer, P. O. , & Bhatta, R. (2021). Analysis of carcass traits and quantitative expression patterns of different meat quality governing genes during heat stress exposure in indigenous goats. Food Chemistry: Molecular Sciences, 3, 100052. 10.1016/j.fochms.2021.100052 35415654 PMC8991526

[fsn33340-bib-0021] Faridah, H. S. , Goh, Y. M. , Noordin, M. M. , & Liang, J. B. (2020). Extrusion enhances apparent metabolizable energy, ileal protein and amino acid digestibility of palm kernel cake in broilers. Asian‐Australasian Journal of Animal Sciences, 33(12), 1965–1974. 32164059 10.5713/ajas.19.0964 PMC7649399

[fsn33340-bib-0022] Froidurot, A. , & Julliand, V. (2022). Cellulolytic bacteria in the large intestine of mammals. Gut Microbes, 14(1), 2031694. 35184689 10.1080/19490976.2022.2031694 PMC8865330

[fsn33340-bib-0023] Güngör, Ö. F. , Özbeyaz, C. , Ünal, N. , Akyüz, H. Ç. , Arslan, R. , & Akçapınar, H. (2022). Evaluation of the genotype and slaughter weight effect on the meat production traits: Comparison of fattening, slaughter, and carcass characteristics between two native sheep. Small Ruminant Research, 217, 106846. 10.1016/j.smallrumres.2022.106846

[fsn33340-bib-0024] Guo, J. R. , Dong, X. F. , Liu, S. , & Tong, J. M. (2018). High‐throughput sequencing reveals the effect of Bacillus subtilis CGMCC 1.921 on the cecal microbiota and gene expression in ileum mucosa of laying hens. Poultry Science, 97(7), 2543–2556. 10.3382/ps/pey112 29897524

[fsn33340-bib-0025] Guo, Y.‐x. , Yang, R.‐c. , Duan, C.‐h. , Yong, W. , Hao, Q.‐h. , Ji, S.‐k. , Yan, H. , Zhang, Y.‐j. , & Liu, Y.‐q. (2022). Effect of Dioscorea opposite waste on growth performance, blood parameters, rumen fermentation and rumen bacterial Community in Weaned Lambs. Journal of Integrative Agriculture, 21(1), 1–24.

[fsn33340-bib-0026] Hammelman, J. E. , Bowker, B. C. , Grant, A. L. , Forrest, J. C. , Schinckel, A. P. , & Gerrard, D. E. (2003). Early postmortem electrical stimulation simulates PSE pork development. Meat Science, 63(1), 69–77. 22061988 10.1016/s0309-1740(02)00057-8

[fsn33340-bib-0027] Han, P. , Li, P. , Zhou, W. , Fan, L. , Wang, B. , Liu, H. , Gao, C. , du, T. , Pu, G. , Wu, C. , Zhang, Z. , Niu, P. , Huang, R. , & Li, H. (2020). Effects of various levels of dietary fiber on carcass traits, meat quality and myosin heavy chain I, IIa, IIx and IIb expression in muscles in Erhualian and large white pigs. Meat Science, 169, 108160. 32593014 10.1016/j.meatsci.2020.108160

[fsn33340-bib-0028] Hao, X. Y. , Yu, S. C. , Mu, C. T. , Wu, X. D. , Zhang, C. X. , Zhao, J. X. , & Zhang, J. X. (2020). Replacing soybean meal with flax seed meal: Effects on nutrient digestibility, rumen microbial protein synthesis and growth performance in sheep. Animal, 14(9), 1841–1848. 32172723 10.1017/S1751731120000397

[fsn33340-bib-0029] Hess, M. , Sczyrba, A. , Egan, R. , Kim, T. W. , Chokhawala, H. , Schroth, G. , Luo, S. , Clark, D. S. , Chen, F. , Zhang, T. , Mackie, R. I. , Pennacchio, L. A. , Tringe, S. G. , Visel, A. , Woyke, T. , Wang, Z. , & Rubin, E. M. (2011). Metagenomic discovery of biomass‐degrading genes and genomes from cow rumen. Science, 331(6016), 463–467. 10.1126/science.1200387 21273488

[fsn33340-bib-0030] Hoa, V.‐B. , Song, D.‐H. , Seol, K.‐H. , Kang, S.‐M. , Kim, H.‐W. , Kim, J.‐H. , & Cho, S. H. (2022). Coating with chitosan containing lauric acid (C12: 0) significantly extends the shelf‐life of aerobically–packaged beef steaks during refrigerated storage. Meat Science, 184, 108696. 34741876 10.1016/j.meatsci.2021.108696

[fsn33340-bib-0031] Hopkins, D. L. , & Ertbjerg, P. (2023). The eating quality of meat: II—Tenderness. In Lawrie's meat science (pp. 393–420). Elsevier.

[fsn33340-bib-0032] Huang, Q. , Dong, K. , Wang, Q. , Huang, X. , Wang, G. , An, F. , Luo, Z. , & Luo, P. (2022). Changes in volatile flavor of yak meat during oxidation based on multi‐omics. Food Chemistry, 371, 131103. 34537608 10.1016/j.foodchem.2021.131103

[fsn33340-bib-0033] Jang, J. C. , Kim, K. H. , Kim, D. H. , Jang, S. K. , Hong, J. S. , Heo, P. S. , & Kim, Y. Y. (2020). Effects of increasing levels of palm kernel meal containing β‐mannanase to growing‐finishing pig diets on growth performance, nutrient digestibility, and pork quality. Livestock Science, 238, 104041. 10.1016/j.livsci.2020.104041

[fsn33340-bib-0034] Jaworski, N. W. , Shoulders, J. , González‐Vega, J. C. , & Stein, H. H. (2014). Effects of using copra meal, palm kernel expellers, or palm kernel meal in diets for weanling pigs. The Professional Animal Scientist, 30(2), 243–251.

[fsn33340-bib-0035] Khan, S. , Khan, R. U. , Sultan, A. , Khan, M. , Hayat, S. U. , & Shahid, M. S. (2016). Evaluating the suitability of maggot meal as a partial substitute of soya bean on the productive traits, digestibility indices and organoleptic properties of broiler meat. Journal of Animal Physiology and Animal Nutrition, 100(4), 649–656. 26847519 10.1111/jpn.12419

[fsn33340-bib-0036] Kirkpatrick, L. T. , Elgin, J. M. , Matarneh, S. K. , Wicks, J. C. , Daniels, R. P. , Yen, C. N. , Bodmer, J. S. , Zumbaugh, M. D. , el‐Kadi, S. W. , Silva, S. L. , Shi, T. H. , & Gerrard, D. E. (2022). Inherent factors influence color variations in semimembranosus muscle of pigs. Meat Science, 185, 108721. 34923395 10.1016/j.meatsci.2021.108721

[fsn33340-bib-0037] Ko, Y. J. , Cha, J. , Jeong, W.‐Y. , Lee, M.‐E. , Cho, B.‐H. , Nisha, B. , Jeong, H. J. , Park, S. E. , & Han, S. O. (2022). Bio‐isopropanol production in Corynebacterium glutamicum: Metabolic redesign of synthetic bypasses and two‐stage fermentation with gas stripping. Bioresource Technology, 354, 127171. 35472638 10.1016/j.biortech.2022.127171

[fsn33340-bib-0038] Krebs, H. A. , & Johnson, W. A. (1980). The role of citric acid in intermediate metabolism in animal tissues. FEBS Letters, 117(S1), K2–K10. 10.4159/harvard.9780674366701.c143 6998725

[fsn33340-bib-0039] Kumar, P. , Lee, J.‐H. , Beyenal, H. , & Lee, J. (2020). Fatty acids as antibiofilm and antivirulence agents. Trends in Microbiology, 28(9), 753–768. 32359781 10.1016/j.tim.2020.03.014

[fsn33340-bib-0040] Lee, W. J. , Koh, E. H. , Won, J. C. , Kim, M.‐S. , Park, J.‐Y. , & Lee, K.‐U. (2005). Obesity: The role of hypothalamic AMP‐activated protein kinase in body weight regulation. The International Journal of Biochemistry & Cell Biology, 37(11), 2254–2259. 16085448 10.1016/j.biocel.2005.06.019

[fsn33340-bib-0041] Li, C. , Yan, F. , Kang, S. , Yan, C. , Hu, Z. , Chen, P. , Gao, S. , Zhang, C. , He, C. , Kaspari, S. , & Stubbins, A. (2021). Carbonaceous matter in the atmosphere and glaciers of the Himalayas and the Tibetan plateau: An investigative review. Environment International, 146, 106281. 33395932 10.1016/j.envint.2020.106281

[fsn33340-bib-0042] Liang, S. , Liu, X. , Zhao, J. , Liu, R. , Huang, X. , Liu, Y. , Yang, X. , & Yang, X. (2022). Effects of high‐dose folic acid on protein metabolism in breast muscle and performance of broilers. Poultry Science, 101, 101935. 10.1016/j.psj.2022.101935 PMC9382563 35961252

[fsn33340-bib-0018] Lima Valença, R. , Silva Sobrinho, A. G. , Romanzini, E. P. , de Andrade, N. , Borghi, T. H. , Zeola, N. M. B. L. , Cirne, L. G. A. , & da Silva Oliveira, V. (2020). Peanut meal and crude glycerin in lamb diets: Meat quality and fatty acid profile. Small Ruminant Research, 185, 106076.

[fsn33340-bib-0043] Liu, D. , Bai, L. , Feng, X. , Chen, Y. P. , Zhang, D. , Yao, W. , Zhang, H. , Chen, G. , & Liu, Y. (2020). Characterization of Jinhua ham aroma profiles in specific to aging time by gas chromatography‐ion mobility spectrometry (GC‐IMS). Meat Science, 168, 108178. 32417671 10.1016/j.meatsci.2020.108178

[fsn33340-bib-0044] Liu, S. , Du, M. , Tu, Y. , You, W. , Chen, W. , Liu, G. , Li, J. , Wang, Y. , Lu, Z. , Wang, T. , & Shan, T. (2022). Fermented mixed feed alters growth performance, carcass traits, meat quality and muscle fatty acid and amino acid profiles in finishing pigs. Animal Nutrition, 12, 87–95. 36632618 10.1016/j.aninu.2022.09.003 PMC9822949

[fsn33340-bib-0045] Lozupone, C. , Knight, R. J. A. , & Microbiology, E. (2005). UniFrac: A new phylogenetic method for comparing microbial. Communities, 71(12), 8228–8235. 10.1128/AEM.71.12.8228-8235.2005 PMC1317376 16332807

[fsn33340-bib-0046] Ma, B. , Zhang, C. , Raza, S. H. A. , Yang, B. , Aloufi, B. H. , Alshammari, A. M. , AlGabbani, Q. , Khan, R. , Hou, S. , & Gui, L. (2022). Effects of dietary non‐fibrous carbohydrate (NFC) to neutral detergent fiber (NDF) ratio change on rumen bacterial community and ruminal fermentation parameters in Chinese black Tibetan sheep (Ovis aries). Small Ruminant Research, 216, 106793.

[fsn33340-bib-0047] Mateos, G. G. , Jiménez‐Moreno, E. , Serrano, M. P. , & Lázaro, R. P. (2012). Poultry response to high levels of dietary fiber sources varying in physical and chemical characteristics. Journal of Applied Poultry Research, 21(1), 156–174.

[fsn33340-bib-0048] McCloskey, D. , Xu, S. , Sandberg, T. E. , Brunk, E. , Hefner, Y. , Szubin, R. , Feist, A. M. , & Palsson, B. O. (2018). Adaptive laboratory evolution resolves energy depletion to maintain high aromatic metabolite phenotypes in Escherichia coli strains lacking the phosphotransferase system. Metabolic Engineering, 48, 233–242. 29906504 10.1016/j.ymben.2018.06.005

[fsn33340-bib-0049] Moreira, L. R. S. (2008). An overview of mannan structure and mannan‐degrading enzyme systems. Applied Microbiology and Biotechnology, 79(2), 165–178. 18385995 10.1007/s00253-008-1423-4

[fsn33340-bib-0050] Newton, K. G. , & Gill, C. O. (1981). The microbiology of DFD fresh meats: A review. Meat Science, 5(3), 223–232. 22056031 10.1016/0309-1740(81)90005-X

[fsn33340-bib-0051] Ng, W.‐K. (2003). The potential use of palm kernel meal in aquaculture feeds. Aquaculture Asia, 8(1), 38–39.

[fsn33340-bib-0052] Nitta, K. , Laviña, W. A. , Pontrelli, S. , Liao, J. C. , Putri, S. P. , & Fukusaki, E. (2019). Metabolome analysis revealed the knockout of glyoxylate shunt as an effective strategy for improvement of 1‐butanol production in transgenic Escherichia coli. Journal of Bioscience and Bioengineering, 127(3), 301–308. 30482596 10.1016/j.jbiosc.2018.08.013

[fsn33340-bib-0053] Olijhoek, D. W. , Hellwing, A. L. F. , Noel, S. J. , Lund, P. , Larsen, M. , Weisbjerg, M. R. , & Børsting, C. F. (2022). Feeding up to 91% concentrate to Holstein and Jersey dairy cows: Effects on enteric methane emission, rumen fermentation and bacterial community, digestibility, production, and feeding behavior. Journal of Dairy Science, 105(12), 9523–9541. 36207184 10.3168/jds.2021-21676

[fsn33340-bib-0054] Ouali, A. , Gagaoua, M. , Boudida, Y. , Becila, S. , Boudjellal, A. , Herrera‐Mendez, C. H. , & Sentandreu, M. A. (2013). Biomarkers of meat tenderness: Present knowledge and perspectives in regards to our current understanding of the mechanisms involved. Meat Science, 95(4), 854–870. 23790743 10.1016/j.meatsci.2013.05.010

[fsn33340-bib-0055] Phulia, V. , Sardar, P. , Sahu, N. P. , Shamna, N. , Fawole, F. J. , Gupta, S. , & Gadhave, P. D. (2017). Replacement of soybean meal with fermented Jatropha curcas kernel meal in the diet of Labeo rohita fingerlings: Effect on hemato‐biochemical and histopathological parameters. Journal of the World Aquaculture Society, 48(4), 676–683.

[fsn33340-bib-0056] Reeves, A. E. , Koenigsknecht, M. J. , Bergin, I. L. , & Young, V. B. (2012). Suppression of Clostridium difficile in the gastrointestinal tracts of germfree mice inoculated with a murine isolate from the family Lachnospiraceae. Infection and Immunity, 80(11), 3786–3794. 22890996 10.1128/IAI.00647-12 PMC3486043

[fsn33340-bib-0057] Ribeiro, R. D. X. , Oliveira, R. L. , Oliveira, R. L. , de Carvalho, G. G. P. , Medeiros, A. N. , Correia, B. R. , Silva, T. M. , & Bezerra, L. R. (2018). Palm kernel cake from the biodiesel industry in diets for goat kids. Part 1: Nutrient intake and utilization, growth performance and carcass traits. Small Ruminant Research, 165, 17–23. 10.1016/j.smallrumres.2018.05.013

[fsn33340-bib-0058] Santos, G.d. O. , Parente, H. N. , Zanine, A. M. , Nascimento, T. V. C. , de Ov Lima, A. G. , Bezerra, L. R. , Machado, N. A. F. , Ferreira, D. J. , dos Santos, V. L. F. , Costa, H. H. A. , Oliveira, J. S. , & Parente, M. O. M. (2022). Effects of dietary greasy babassu byproduct on nutrient utilization, meat quality, and fatty acid composition in abomasal digesta and meat from lambs. Animal Feed Science and Technology, 287, 115283.

[fsn33340-bib-0059] Santos‐Silva, J. , Alves, S. P. , Francisco, A. , Portugal, A. P. , Almeida, J. , Fialho, L. , Jerónimo, E. , & Bessa, R. J. B. (2020). Effects of a high‐fibre and low‐starch diet in growth performance, carcass and meat quality of young Alentejana breed bulls. Meat Science, 168, 108191. 32450454 10.1016/j.meatsci.2020.108191

[fsn33340-bib-0060] Saxton, R. A. , & Sabatini, D. M. (2017). mTOR signaling in growth, metabolism, and disease. Cell, 168(6), 960–976. 28283069 10.1016/j.cell.2017.02.004 PMC5394987

[fsn33340-bib-0061] Shang, Q. , Liu, S. , Liu, H. , Mahfuz, S. , & Piao, X. (2021). Impact of sugar beet pulp and wheat bran on serum biochemical profile, inflammatory responses and gut microbiota in sows during late gestation and lactation. Journal of Animal Science and Biotechnology, 12(1), 1–14. 33879267 10.1186/s40104-021-00573-3 PMC8059298

[fsn33340-bib-0017] Silva, L. O. , Carvalho, G. G. P. , Tosto, M. S. L. , Lima, V. G. O. , Cirne, L. G. A. , dos Santos Pina, D. , Santos, S. A. , Rodrigues, C. S. , Ayres, M. C. C. , & Azevedo, J. A. G. (2020). Digestibility, nitrogen metabolism, ingestive behavior and performance of feedlot goats fed high‐concentrate diets with palm kernel cake. Livestock Science, 241, 104226.

[fsn33340-bib-0062] Silva, R. V. M. M. , de Carvalho, G. G. P. , Pires, A. J. V. , Pereira, M. L. A. , Pereira, L. , Campos, F. S. , Perazzo, A. F. , de Araújo, M. L. G. M. L. , de Oliveira Nascimento, C. , Santos, S. A. , Tosto, M. S. L. , de Almeida Rufino, L. M. , & de Carvalho, B. M. A. (2016). Cottonseed cake in substitution of soybean meal in diets for finishing lambs. Small Ruminant Research, 137, 183–188.

[fsn33340-bib-0063] Song, Y. , Malmuthuge, N. , Steele, M. A. , & Guan, L. L. (2018). Shift of hindgut microbiota and microbial short chain fatty acids profiles in dairy calves from birth to pre‐weaning. FEMS Microbiology Ecology, 94(3), fix179. 10.1093/femsec/fix179 29267960

[fsn33340-bib-0064] Su, Y. , Sun, X. , Zhao, S. , Hu, M. , Li, D. , Qi, S. , Jiao, X. , Sun, Y. , Wang, C. , Zhu, X. , Li, Z. , & Shi, Y. (2022). Dietary alfalfa powder supplementation improves growth and development, body health, and meat quality of Tibetan sheep. Food Chemistry, 396, 133709. 35872497 10.1016/j.foodchem.2022.133709

[fsn33340-bib-0065] Sundu, B. , Kumar, A. , & Dingle, J. (2006). Palm kernel meal in broiler diets: Effect on chicken performance and health. World's Poultry Science Journal, 62(2), 316–325.

[fsn33340-bib-0066] Vacca, M. , Celano, G. , Calabrese, F. M. , Portincasa, P. , Gobbetti, M. , & De Angelis, M. (2020). The controversial role of human gut lachnospiraceae. Microorganisms, 8(4), 573. 32326636 10.3390/microorganisms8040573 PMC7232163

[fsn33340-bib-0067] Vasta, V. , & Priolo, A. (2006). Ruminant fat volatiles as affected by diet. A Review. Meat Science, 73(2), 218–228. 22062292 10.1016/j.meatsci.2005.11.017

[fsn33340-bib-0068] Wang, B. , Zhao, X. , Zhang, B. , Cui, Y. , Nueraihemaiti, M. , Kou, Q. , & Luo, H. (2022). Assessment of components related to flavor and taste in tan‐lamb meat under different silage‐feeding regimens using integrative metabolomics. Food Chemistry: X, 14, 100269. 35252839 10.1016/j.fochx.2022.100269 PMC8892073

[fsn33340-bib-0069] Wang, F. , Gao, Y. , Wang, H. , Xi, B. , He, X. , Yang, X. , & Li, W. (2021). Analysis of volatile compounds and flavor fingerprint in Jingyuan lamb of different ages using gas chromatography–ion mobility spectrometry (GC–IMS). Meat Science, 175, 108449. 33550158 10.1016/j.meatsci.2021.108449

[fsn33340-bib-0070] Wang, Q. , Li, X. , Xue, B. , Wu, Y. , Song, H. , Luo, Z. , Shang, P. , Liu, Z. , & Huang, Q. (2022). Low‐salt fermentation improves flavor and quality of sour meat: Microbiology and metabolomics. LWT, 171, 114157.

[fsn33340-bib-0071] Warner, R. , Wheeler, T. L. , Ha, M. , Li, X. , Bekhit, A. E.‐D. , Morton, J. , Vaskoska, R. , Dunshea, F. R. , Liu, R. , Purslow, P. , & Zhang, W. (2021). Meat tenderness: Advances in biology, biochemistry, molecular mechanisms and new technologies. Meat Science, 185, 108657–108711. 34998162 10.1016/j.meatsci.2021.108657

[fsn33340-bib-0072] Wen, J. , Zhang, J.‐Q. , Nie, Z.‐L. , Zhong, Y. , & Sun, H. (2014). Evolutionary diversifications of plants on the Qinghai‐Tibetan plateau. Frontiers in Genetics, 5, 4. 24575120 10.3389/fgene.2014.00004 PMC3921583

[fsn33340-bib-0073] Youssef, N. , Sheik, C. S. , Krumholz, L. R. , Najar, F. Z. , Roe, B. A. , & Elshahed, M. S. J. A. (2009). Comparison of species richness estimates obtained using nearly complete fragments and simulated pyrosequencing‐generated fragments in 16S rRNA gene‐based environmental surveys. Applied and Environmental Microbiology, 75(16), 5227–5236. 19561178 10.1128/AEM.00592-09 PMC2725448

[fsn33340-bib-0074] Zhang, M. , Guo, Y. , Su, R. , Corazzin, M. , Hou, R. , Xie, J. , Zhang, Y. , Zhao, L. , Su, L. , & Jin, Y. (2022). Transcriptome analysis reveals the molecular regulatory network of muscle development and meat quality in Sunit lamb supplemented with dietary probiotic. Meat Science, 194, 108996. 36195032 10.1016/j.meatsci.2022.108996

[fsn33340-bib-0075] Zhang, X. , Han, L. , Hou, S. , Raza, S. H. A. , Gui, L. , Sun, S. , Wang, Z. , Yang, B. , Yuan, Z. , Simal‐Gandara, J. , el‐Shehawi, A. M. , Alswat, A. , Alenezi, M. A. , Shukry, M. , Sayed, S. M. , & Aloufi, B. H. (2022). Metabolomics approach reveals high energy diet improves the quality and enhances the flavor of black Tibetan sheep meat by altering the composition of rumen microbiota. Frontiers in Nutrition, 9, 1618–1638. 10.3389/fnut.2022.915558 PMC9405419 36034898

[fsn33340-bib-0076] Zhang, X. , Han, L. , Hou, S. , Raza, S. H. A. , Wang, Z. , Yang, B. , Sun, S. , Ding, B. , Gui, L. , Simal‐Gandara, J. , Shukry, M. , Sayed, S. M. , & al Hazani, T. M. I. (2022). Effects of different feeding regimes on muscle metabolism and its association with meat quality of Tibetan sheep. Food Chemistry, 374, 131611–131624. 34863603 10.1016/j.foodchem.2021.131611

[fsn33340-bib-0077] Zhao, C. , Wang, L. , Ke, S. , Chen, X. , Kenéz, Á. , Xu, W. , Wang, D. , Zhang, F. , Li, Y. , Cui, Z. , Qiao, Y. , Wang, J. , Sun, W. , Zhao, J. , Yao, J. , Yu, Z. , & Cao, Y. (2022). Yak rumen microbiome elevates fiber degradation ability and alters rumen fermentation pattern to increase feed efficiency. Animal Nutrition, 11, 201–214. 36263411 10.1016/j.aninu.2022.07.014 PMC9556794

