# Supplementary material for: Exploring the effects of palm kernel meal feeding on the meat quality and rumen microorganisms of Qinghai Tibetan sheep
Source: Food Sci Nutr. 2023 Apr 5;11(6):3516–34. doi: 10.1002/fsn3.3340 (PMC10261763; doi:10.1002/fsn3.3340)
Supplement: Supplementary file 1 — Figure S1. Figure S2. Table S1. Table S2. Table S3. [file FSN3-11-3516-s001.docx]

**Supplementary figures and Supplementary tables**

**Fig. S1.** Score plot of orthogonal partial least squares discriminant analysis (OPLS-DA) model obtained of ZL-0 and ZL-18, Positive ion mode(A) and Negative ion mode(B).


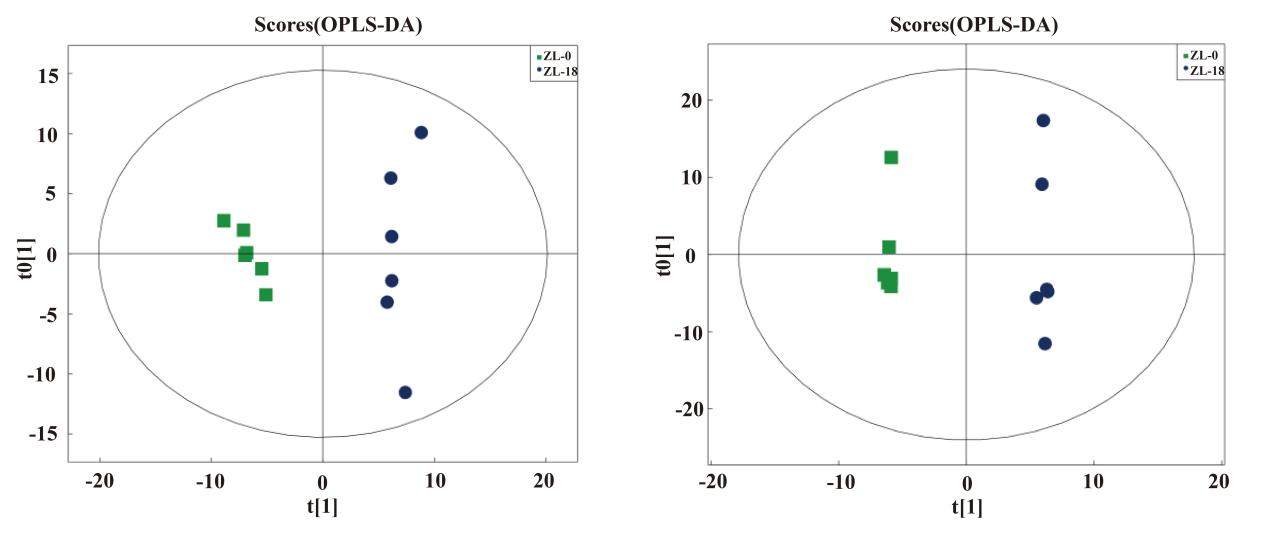


A

B


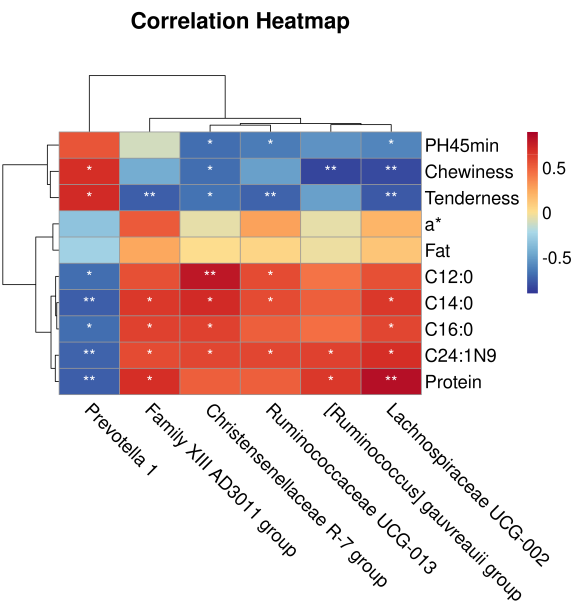

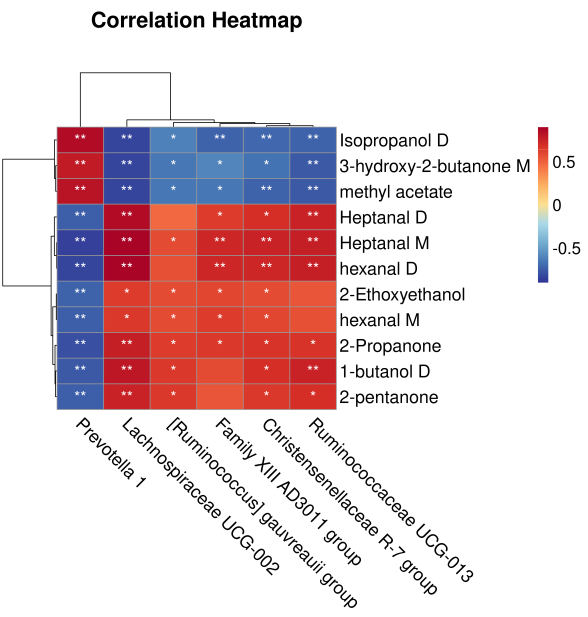


A

B

**Fig. S2.** Pearson’s correlations between different rumen microbiomes and meat quality(A), Volatile flavor components (B). The significant correlations (r > 0.52 or r < −0.52, p < 0.01) were shown in the correlation heatmaps. The color intensity and circle size are proportional to the correlation values.

**Table S1** Effects of different levels of palm meal added in feed on the AA profiles of Tibetan sheep meat (umol/g)

| **Index** | | **ZL-0** | | **Zl-15** | | **Zl-18** | | **Zl-21** | |
| --- | --- | --- | --- | --- | --- | --- | --- | --- | --- |
| glutamate | | 0.23±0.14 | | 0.13±0.04 | | 0.21±0.13 | | 0.15±0.01 | |
| glycine | | 2.75±0.80 | | 2.45±0.48 | | 2.62±0.16 | | 2.53±0.59 | |
| lysine | | 0.14±0.01 | | 0.14±0.02 | | 0.12±0.03 | | 0.12±0.02 | |
| aspartate | | 0.05±0.02 | | 0.02±0.00 | | 0.02±0.00 | | 0.02±0.02 | |
| arginine | | 0.47±0.10 | | 0.40±0.03 | | 0.39±0.01 | | 0.37±0.10 | |
| serine | | 0.38±0.04 | | 0.41±0.11 | | 0.37±0.07 | | 0.42±0.04 | |
| methionine | | 0.08±0.01 | | 0.07±0.01 | | 0.07±0.01 | | 0.08±0.00 | |
| phenylalanine | | 0.19±0.04 | | 0.16±0.01 | | 0.17±0.02 | | 0.18±0.02 | |
| tyrosine | | 0.22±0.05 | | 0.20±0.03 | | 0.19±0.02 | | 0.20±0.01 | |
| leucine | | 0.34±0.02 | | 0.34±0.02 | | 0.34±0.03 | | 0.34±0.02 | |
| Isoleucine | | 0.19±0.02 | | 0.19±0.02 | | 0.19±0.02 | | 0.18±0.00 | |
| histidine | | 1.38±0.36 | | 1.20±0.07 | | 1.25±0.19 | | 1.14±0.09 | |
| proline | | 0.34±0.03 | | 0.31±0.02 | | 0.30±0.04 | | 0.27±0.04 | |
| valine | | 0.46±0.01 | | 0.46±0.03 | | 0.40±0.04 | | 0.38±0.06 | |
| threonine | | 0.32±0.02 | | 0.34±0.07 | | 0.34±0.10 | | 0.33±0.02 | |
| alanine/sarcosine | | 5.07±0.34 | | 4.41±0.65 | | 4.94±0.67 | | 4.52±0.59 | |
| asparagine | | 0.15±0.01 | | 0.17±0.05 | | 0.15±0.05 | | 0.15±0.02 | |
| creatine | | 8.35±1.20 | | 8.40±0.28 | | 8.74±0.37 | | 8.37±0.86 | |
| citrulline | | 0.10±0.04 | | 0.13±0.06 | | 0.14±0.08 | | 0.15±0.12 | |
| Glutamine | | 6.09±3.93 | | 3.92±1.42 | | 4.36±0.59 | | 3.42±0.22 | |
| creatinine | | 0.11±0.01 | | 0.11±0.00 | | 0.12±0.01 | | 0.10±0.01 | |
| tryptophan | | 0.40±0.01 | | 0.40±0.06 | | 0.41±0.06 | | 0.33±0.04 | |
| hydroxyproline | | 0.17±0.05 | | 0.15±0.05 | | 0.16±0.05 | | 0.15±0.04 | |
| ornithine | | 0.11±0.13 | | 0.07±0.02 | | 0.08±0.02 | | 0.06±0.01 | |
| taurine | | 4.64±2.96 | | 5.11±0.60 | | 5.86±1.67 | | 5.26±0.08 | |
| Cystine | | 0.21±0.09 | | 0.22±0.03 | | 0.23±0.02 | | 0.22±0.04 | |
| choline | | 4.96±0.80 | | 5.38±1.54 | | 4.03±0.51 | | 3.31±1.07 | |
| aminoadipic acid | | 0.08±0.07 | | 0.05±0.03 | | 0.05±0.02 | | 0.04±0.00 | |

Only amino acids with a significant difference in at least one of the comparisons were presented.

**Table S2** The detailed results of differential metabolites in the longissimus lumborum in the negative ion detection mode (OPLS-DA VIP > 1 and P value < 0.05).

| Name | Adduct | m/z | Rt(s) | VIP | FC | Variation | P-value |
| --- | --- | --- | --- | --- | --- | --- | --- |
| Linoleoylcarnitine | [M+H]+ | 376.26 | 34.56 | 2.22 | 5.91 | ↑ | 0.00 |
| Oleana-1,9(11)-dien-28-oic acid, 2-cyano-3,12-dioxo-, methyl ester | [M+H]+ | 393.28 | 34.42 | 1.45 | 4.94 | ↑ | 0.00 |
| 3-[(cholamidopropyl)dimethylammonio]-1-propanesulfonate | [M+H-H2O]+ | 178.08 | 296.65 | 3.17 | 5.67 | ↑ | 0.00 |
| (r)-(+)-arachidonyl-1'-hydroxy-2'-propylamide | [M+H-C16H21NO4]+ | 194.12 | 213.25 | 1.35 | 2.17 | ↑ | 0.00 |
| Anserine | [M+H]+ | 118.09 | 396.20 | 1.02 | 1.81 | ↑ | 0.00 |
| Phytosphingosine | [M+H]+ | 180.10 | 232.02 | 2.16 | 6.71 | ↑ | 0.00 |
| DL-Glutamic acid | [M+H-3H2O]+ | 363.29 | 36.14 | 1.11 | 2.45 | ↓ | 0.00 |
| Thebaine | [M+H]+ | 400.34 | 177.44 | 6.23 | 3.16 | ↑ | 0.00 |
| Oleoyl-l-carnitine | [M+H]+ | 416.34 | 200.91 | 4.79 | 3.54 | ↓ | 0.00 |
| Palmitamide | [M+H]+ | 480.34 | 189.75 | 4.13 | 1.92 | ↓ | 0.00 |
| Oleamide | [M+H]+ | 258.11 | 387.14 | 11.21 | 0.42 | ↓ | 0.00 |
| L-palmitoylcarnitine | [M+H]+ | 162.11 | 367.06 | 24.86 | 1.27 | ↑ | 0.00 |
| Diosgenin | [M+H]+ | 126.02 | 304.39 | 10.41 | 0.50 | ↑ | 0.00 |
| 3-hydroxyoleylcarnitine | [M+H]+ | 279.08 | 416.51 | 2.89 | 1.47 | ↑ | 0.00 |
| Lpc 18:1 | [M+H]+ | 426.36 | 174.07 | 7.93 | 2.20 | ↓ | 0.01 |
| 3-hydroxyhexadecanoylcarnitine | (M+CH3CN+H)+ | 298.15 | 447.83 | 1.32 | 3.63 | ↑ | 0.01 |
| N-cyclohexylaniline | [M+H-H2O]+ | 313.27 | 33.56 | 1.77 | 1.17 | ↑ | 0.01 |
| Trimethylamine | [M+H]+ | 156.08 | 457.07 | 2.74 | 1.28 | ↑ | 0.01 |
| 1,2-dipentadecanoyl-sn-glycero-3-phosphocholine | [M+H]+ | 255.14 | 445.04 | 5.45 | 2.13 | ↓ | 0.01 |
| Cis-13-eicosenoic acid | [M+H]+ | 703.57 | 183.75 | 4.64 | 1.40 | ↑ | 0.01 |
| Oleoyl ethylamide | (M+NH4)+ | 684.25 | 489.53 | 1.21 | 0.04 | ↓ | 0.01 |
| Demeton s | [M+H]+ | 527.15 | 453.84 | 1.19 | 0.03 | ↑ | 0.01 |
| DL-arginine | [M+H-H2O]+ | 369.35 | 32.91 | 2.57 | 1.61 | ↑ | 0.01 |
| 1,2-dipalmitoleoyl-sn-glycero-3-phosphocholine | [M+H]+ | 788.61 | 41.56 | 4.55 | 0.80 | ↓ | 0.01 |
| 1-palmitoyl-sn-glycero-3-phosphocholine | [M+NH4]+ | 360.15 | 397.74 | 2.06 | 0.10 | ↓ | 0.01 |
| 2-amino-5-chlorobenzoxazole | [M+H-C2H4]+ | 283.02 | 471.55 | 3.71 | 0.54 | ↑ | 0.02 |
| 7-oxabicyclo[2.2.1]heptane | [M+H]+ | 344.28 | 187.75 | 1.97 | 3.24 | ↓ | 0.02 |
| DL-tyrosine | [M+NH4]+ | 442.35 | 196.94 | 4.15 | 2.09 | ↑ | 0.02 |
| Phosphocreatine | [M+H]+ | 240.10 | 227.81 | 3.02 | 0.20 | ↑ | 0.02 |
| Lpc 18:2 | [M+H-CH5ON3]+ | 116.07 | 324.53 | 2.37 | 0.70 | ↓ | 0.02 |
| Lauroyl-l-carnitine | [M+H-H2O]+ | 321.31 | 34.31 | 1.37 | 0.56 | ↑ | 0.02 |
| Trimethylamine n-oxide | [M+H]+ | 218.11 | 423.71 | 1.90 | 1.40 | ↑ | 0.02 |
| 1-hexadecyl-2-(8z,11z,14z-eicosatrienoyl)-sn-glycero-3-phosphocholine | [M+H]+ | 424.34 | 175.67 | 2.62 | 1.55 | ↑ | 0.02 |
| Sulfallate | [M+H]+ | 731.61 | 181.88 | 5.48 | 1.51 | ↑ | 0.02 |
| Decanoyl-l-carnitine | [M+Na]+ | 808.58 | 40.53 | 2.65 | 0.83 | ↓ | 0.03 |
| 1-ethylpiperazine | [M+H]+ | 442.35 | 160.85 | 1.29 | 1.75 | ↓ | 0.03 |
| Linoleoylglycine | [M+H]+ | 792.59 | 40.13 | 1.91 | 1.40 | ↓ | 0.03 |

**Table S3.** Evaluation Parameters of Positive and Negative Ion Mode OPLS-DA Model

| Type | R^2^X(cum) | R^2^Y(cum) | Q^2^(cum) | Title |
| --- | --- | --- | --- | --- |
| Positive |  |  |  |  |
| OPLS-DA | 0.284 | 0.972 | 0.493 | ZL-0_VS_ZL-18 |
| Negative |  |  |  |  |
| OPLS-DA | 0.555 | 0.998 | 0.215 | ZL-0_VS_ZL-18 |
